# Maternal Microbiota Transfer Programs Offspring Eating Behavior

**DOI:** 10.3389/fmicb.2021.672224

**Published:** 2021-06-15

**Authors:** Anne-Lise Pocheron, Gwenola Le Dréan, Helene Billard, Thomas Moyon, Anthony Pagniez, Christine Heberden, Emmanuelle Le Chatelier, Dominique Darmaun, Catherine Michel, Patricia Parnet

**Affiliations:** ^1^UN, INRAE, UMR 1280, PhAN, IMAD, Nantes, France; ^2^INRAE, Micalis, Jouy-en-Josas, France; ^3^INRAE, MetaGenoPolis, Jouy en-Josas, France

**Keywords:** 16S rDNA sequencing, food preference, motivation, obesity, DOHaD (development origins of health and disease), OP rat

## Abstract

Understanding the link between mother’s obesity and regulation of the child’s appetite is a prerequisite for the design of successful preventive strategies. Beyond the possible contributions of genetic heritage, family culture, and hormonal and metabolic environment during pregnancy, we investigate in the present paper the causal role of the transmission of the maternal microbiotas in obesity as microbiotas differ between lean and obese mothers, maternal microbiotas are the main determinants of a baby’s gut colonization, and the intestinal microbiota resulting from the early colonization could impact the feeding behavior of the offspring with short- and long-term consequences on body weight. We thus investigated the potential role of vertical transfers of maternal microbiotas in programming the eating behavior of the offspring. Selectively bred obese-prone (OP)/obese-resistant (OR) Sprague-Dawley dams were used since differences in the cecal microbiota have been evidenced from males of that strain. Microbiota collected from vagina (at the end of gestation), feces, and milk (at postnatal days 1, 5, 10, and 15) of OP/OR dams were orally inoculated to conventional Fischer F344 recipient pups from birth to 15 days of age to create three groups of pups: F-OP, F-OR, and F-Sham group (that received the vehicle). We first checked microbiotal differences between inoculas. We then assessed the impact of transfer (from birth to adulthood) onto the intestinal microbiota of recipients rats, their growth, and their eating behavior by measuring their caloric intake, their anticipatory food reward responses, their preference for sweet and fat tastes in solutions, and the sensations that extend after food ingestion. Finally, we searched for correlation between microbiota composition and food intake parameters. We found that maternal transfer of microbiota differing in composition led to alterations in pups’ gut microbiota composition that did not last until adulthood but were associated with specific eating behavior characteristics that were predisposing F-OP rats to higher risk of over consuming at subsequent periods of their life. These findings support the view that neonatal gut microbiotal transfer can program eating behavior, even without a significant long-lasting impact on adulthood microbiota composition.

## Introduction

Obesity remains a major public health concern since its prevalence is still on the rise in specific age groups, particularly in the 5–19-year-old group (Who, 2020), and a third of French women of childbearing age are either overweight or obese (Matta et al., 2016). Obesity in this age range is of particular concern since there is overwhelming evidence that being born to an obese mother increases the risk for the child to develop excess adiposity (Weng et al., 2013; Juonala et al., 2020). Birth weight and weight gain during infancy are important factors in the subsequent development of obesity and chronic diseases associated with excess of adiposity, such as type 2 diabetes or cardiovascular disease The GBD 2015 Obesity Collaborators, 2017, which expose the future adult to a higher risk of death (Abdelaal et al., 2017). Excess intake of calories and alteration of the reward system regulation, associated with low physical activity and consumption of food of low quality, are among the main determinants of the vast majority of cases of obesity not associated to genetic polymorphism. Understanding the link between regulation of the child’s appetite and his/her mother’s obesity is a prerequisite for the design of successful preventive strategies. We propose in the present paper to investigate the causal role of transmission of maternal microbiotas, in obesity, as microbiotas differ between lean and obese mothers, are the main determinants of offspring gut colonization, and the resulting intestinal microbiota could impact feeding behavior of the offspring.

Most studies comparing the fecal microbiotas of obese or overweight subjects with lean ones concluded that there were differences in composition (for review, see Gerard, 2016). Such differences were reported in pregnant women (Collado et al., 2008; Dreisbach et al., 2020) regarding not only intestinal microbiota but also the microbiota associated with breast milk (Cabrera-Rubio et al., 2012; Lundgren et al., 2019), and vaginal mucosa (Si et al., 2017). Despite some discrepancies (e.g., Stanislawski et al., 2017), differences were also found among descendants depending on whether they were born to obese or lean mothers (for review, see Dreisbach et al., 2020). Such a finding is consistent with the major contribution of maternal microbiotas to the initial colonization of the infant’s gastrointestinal tract (Ferretti et al., 2018). Bacterial colonization of the human sterile gut begins during passage though the birth canal and undergoes distinct phases of progression during the first months of life to reach a richness and diversity almost similar to that of adults by 3 years of age, even though complete maturation of the gut microbiota might take longer (Stewart et al., 2018; Derrien et al., 2019). Bacterial strains tracking between maternal sources and infant gut microbiotas has evidenced that all maternal microbiotas provide inocula for this colonization process with vertical transfers demonstrated for vaginal bacteria (Matsumiya et al., 2002), fecal bacteria (Backhed et al., 2015; Asnicar et al., 2017; Ferretti et al., 2018), as well as for bacteria associated to breast milk (Martin et al., 2012; Jost et al., 2014; Pannaraj et al., 2017). Among these strains, some were only transiently detected, particularly for species typical of the vaginal microbiota which become undetectable by 1 week of age (Ferretti et al., 2018).

Because of its vast biochemical potential superimposed on that of the host, the gut microbiota can contribute to the host’s phenotype, both by interacting with its organ functions in adulthood or by programming organ development in the infant. Previously, the gut microbiota has been shown to be involved in improving the energy yields from food, or modulating energy balance (for review, see Heiss and Olofsson, 2018), or in the accretion of the adipose tissue in animal models (Backhed et al., 2004) and humans (Le Roy et al., 2019). However, microbial communities are also increasingly recognized as modulators of complex animal behaviors such as social interactions, stress-induced behavior (anxiety, depression), as well as cognitive behavior (learning and memory tasks) (for review, see Vuong et al., 2017). The contribution of gut microbiota in feeding behavior (appetite or eating regulation) has been first evidenced by demonstrating differences in food intake between germ-free and conventional animals (Backhed et al., 2004; Rabot et al., 2010) and a decrease in food intake following the intraperitoneal injections of intestinal *Escherichia coli* ClpB protein in mice (Breton et al., 2016). Such contribution is supported by the fact that germ-free status is associated with an altered expression of several genes involved in food intake regulation, such as *bdnf* or *gcg* in brain (Diaz Heijtz et al., 2011; Schele et al., 2013)or the hypothalamic inhibitory factor receptor GABA-R, whose expression is restored by supplementation of a particular bacterial strain (*Lactobacillus rhamnosus*, strain JB-1) (Bravo et al., 2011). Lastly, prebiotic supplementation has been shown to modify the balance between limbic and hypothalamic gene expression involved in food reward and food intake (Delbes et al., 2018). However, the ability of early gut microbiota to program the eating behavior of adults is poorly studied (van de Wouw et al., 2017). This issue clearly deserves further study given the large body of evidence showing that eating behavior and the neural circuits involved in its control, i.e., the vagus nerve, hypothalamus, and reward circuits, can be programmed early in life through nutrition and the environment factors (Coupe et al., 2010; Ross and Desai, 2014; Ndjim et al., 2017; Paradis et al., 2017).

We therefore investigated the potential role of microbiota transfer from mother to child as a route through which the eating behavior of the offspring can be programmed.

To test such hypothesis, we used selectively bred obese-prone (OP)/obese-resistant (OR) Sprague-Dawley rats. Of particular relevance to our hypothesis, males from these strains exhibit differences in their cecal microbiota (Obanda et al., 2018). We collected feces-, vagina-, and milk-associated microbiota from OP/OR dams before transferring them to conventional Fischer F344 recipient pups between birth to 15 days of age, so as to form three groups of pups: F-OP, F-OR, and F-Sham groups (that received the vehicle). As a prerequisite, we checked that inoculas differed in their microbiotal composition according to the genetic background of the donors and assessed the impact of transfer onto the gut microbiota of recipient rats at PND21, PND60, PND130, and PND200. We then characterized the growth and eating behavior of the three groups, from birth to adulthood, by measuring their calorie intake, their anticipatory food reward responses, the taste perception or gustation during ingestion of sweet and fat solution, and the sensations that extend after food ingestion. We finally searched for correlations between microbiota composition and food intake parameters.

## Materials and Methods

### Ethics Statement

All experiments were conducted in accordance with the European Union regulations for the care and use of animals for experimental procedures (2010/63/EU). Protocols were approved by the local Committee on the Ethics in Animal Experiments of Pays de la Loire (France) and the French Ministry of Research (APAFIS#6617-2016072916395797-v6). Animal facility is registered by the French Veterinary Department as A44276.

### Inocula

#### Donor Mothers

**FIGURE 1:**
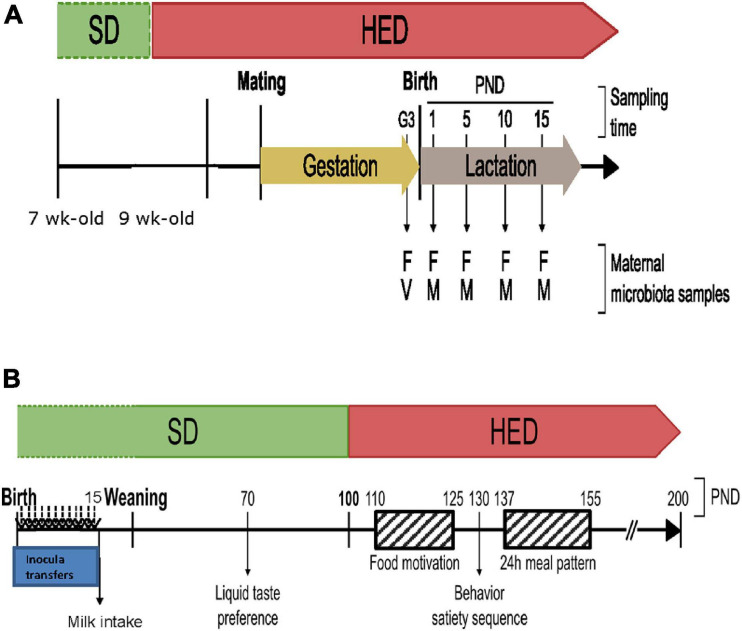
**(A)** Timeline of maternal microbiota collection from OP and OR dams to produce inocula. Vaginal microbiota (V) was collected at the end of the third week of gestation (G3). Milk microbiota (M) was collected every 5 days from postnatal day (PND)1 to PND15. Fecal microbiota (F) was collected at every time point. **(B)** Inocula transfers and timeline of behavioral tests performed on Fischer pups before and after weaning.

Obese-prone [Crl:OP(CD), OP] and obese-resistant [Crl:OR(CD), OR] 7-week-old female rats derived from those initially selected by Levin (Levin et al., 1997) were obtained from Charles River Labs (Kingston, NY, United States). Following 2 weeks adaptation to a standard diet (A03, Safe Diet, Augy, France), females were fed a high energetic diet (HED) (58V8, TestDiet, Richmond, United States) (for more details, see Supplementary Table 2) for 4 weeks then mated with Crl:OR(CD) or standard Sprague-Dawley (RjHan:SD) male breeders for OR and OP females, respectively. Six OP and six OR completed gestations and lactations after mating (Figure 1A).

#### Microbiota Sampling

Feces were collected 2 to 3 days before parturition (G3) and on days 5, 10, and 20 of lactation (Figure 1A). For vaginal and milk sampling, dams were anesthetized with isoflurane 2.5% + dioxygen (5 L/min) and shaved. Their ventral and anogenital area were carefully cleaned with 70% ethanol to ensure sterility. Vaginal smears were sampled around 2–3 days before parturition (G3) using a sterile swab (Puritan HydraFlock, Guilford, United States) humidified with sterile 0.9% NaCl and collected in a 2-ml sealed sterile tube (Sarstedt, Nümbrecht, Germany) containing 1 ml of 0.9% NaCl. Milk was collected as previously described (Martin Agnoux et al., 2015), at days 1, 5, and 10 of lactation (Figure 1A). Briefly, dams were injected intraperitoneally with 0.3 ml of oxytocin (Syntocinon, Sigma-Tau, Ivry-sur-Seine, France) (5 UI/ml) 15 min before anesthesia then milk was manually collected with sterile chirurgical gloves (Gammex Latex Ansell, Melaka, Malaysia) and sterile cottoned glass Pasteur pipette.

#### Inocula Preparation

Protocols for inocula preparation were chosen considering both feasibility and the need to mimic physiological conditions. To be close to the natural supply of milk-associated bacteria in one suckling [as calculated from our unpublished bacteria enumeration from rat milk and published data about milk consumption by rat pups (Bautista et al., 2008; Sevrin et al., 2017)], we had to deliver milk-derived inocula after bacterial cultivation. To mimic transmissions of fecal exchange between dams and pups, we inoculated 10^8^ fecal bacteria per pup and per day.

Vaginal-, fecal-, and milk-derived inocula were prepared from the above microbiotal samples as detailed in Supplementary Material and Methods from OP and OR donors and were pooled 2 × 2 for each genotype in order to obtain enough material for carrying out microbiota transfer to offspring in duplicate experiments.

### Maternal Microbiota Transfer Experiment

Primiparous and conventional female Fischer (F344/HanZtmRj) rats (*n* = 18) were obtained at 1-day of gestation (G1) from Janvier-labs (Le Genest Saint Isle, France) and housed individually (22°C ± 2°C, 12:12-h reversed light/dark cycle) with free access to tap water and standard diet (A03, Safe Diet, Augy, France). This experimental protocol was carried on a total number of six litters for each experimental group during two independent sessions. The overall design of the study was as follows: for each dam, litter was culled to eight pups (four males and four females) that received as soon as possible the various inocula. For each session, three litters received the pools of inocula prepared from three pairs of OP dams (F-OP group), three litters received the pools of inocula prepared from three pairs of OR dams (F-OR group), and three litters received stock solution (F-Sham group). Pups’ weight was monitored every day until postnatal day 15 (PND15) that corresponded to the end of microbiota transfers (Figure 1B). Pups were weaned at PND21 and housed individually (22°C ± 2°C, 12:12-h reversed light/dark cycle) with free access to tap water and standard diet A03 (Safe Diet, Augy, France). They were then fed with the HED (58V8, TestDiet, Richmond, United States) from PND100 to the end of the experiment. Weight and food intake were regularly monitored from PND21 to PND200. Feces were harvested at PND60 and PND130.

All inocula were thawed at room temperature and gently transferred to pups by spontaneous oral intake using a micropipette. Vaginal inocula were transferred (5.0 to 5.7 log eq Bacteria per pup, Supplementary Table 1) first and only once within 2 h after birth (PND0) while both fecal and milk-derived inocula were administered daily, at the same time of day, from PND0 to PND15. For the latter, the volumes transferred were adapted as pups grew, resulting in a slight increase in the bacterial load (from 3.2 to 6.6 log bacteria per pup, Supplementary Table 1).

### Cecocolonic and Cecal Contents

After death by intracardiac injection of 0.5 ml of Exagon^®^ (Richter Pharma, Wels, Austria), the cecocolonic content of one male and one female pup was collected in each litter and pooled at PND11 (*n* = 6 per group). At PND20/21, cecocolonic contents were collected from three females/six males of the first session and six females/three males of the second one, in order to reach a number of nine rats per group and sex. Final numbers may differ from those expected due to sexing errors at birth. At PND20/21, the sizes of groups were as follows for females: F-Sham *n* = 10, F-OP *n* = 8, and F-OR *n* = 9; for males: F-Sham *n* = 8, F-OP *n* = 10, and F-OP *n* = 9. At adulthood, i.e., PND200/201 for the first session and PND196/197 for the second one, cecal contents were collected from three males/six females and six males/three females, respectively. Final effective were for females: F-Sham *n* = 9, F-OP *n* = 9, and F-OR *n* = 7; for males: F-Sham *n* = 9, F-OP *n* = 9, and F-OR *n* = 11. Contents were weighted and mixed in three times their volume in sterile water. After complete homogenization, these cecocolic/cecal suspensions were centrifuged (20 min, 7,800 × *g*, 4°C) then both supernatants and pellets were frozen at −20°C for microbiota analysis, respectively.

### Microbiotal Characterization of Inocula and Intestinal Contents From F344 Recipients

#### DNA Extraction

DNA was extracted from inocula and from intestinal or fecal samples using kits from Qiagen, (Hilden, Germany) after enzymatic and possibly mechanical lysis according to protocols specifically adapted to the nature of the samples (see Supplementary Material and Methods).

#### DNA Quantification and Total Bacteria Count by qPCR

DNA concentrations in extracts were quantified using a NanoVue^TM^ spectrophotometer (GE Healthcare, Little Chalfont, Bucks, United Kingdom).

Total bacteria counts were enumerated on DNA extracts using real-time quantitative polymerase chain reactions (qPCR). The analysis was performed using conditions described in Fanca-Berthon et al. (2010) with the following forward and reverse primers: 5′-WCCTACGGGWGGCAGCAGTS-3′; 5′-TTACCGCGGCTGCTGGCACR-3′.

#### 16S rDNA Sequencing

The V4 hypervariable region of the 16S rDNA gene was amplified from the DNA extracts by PCR using composite primers: 5′-CTT TCCCTACACGACGCTCTTCCGATCTGTGYCAGCMGCCGC GGTAA-3′ and 5′-GGAGTTCAGACGTGTGCTCTTCCGATCT GGACTACHVGGGTWTCTAAT-3′ which were based on the primers adapted from Caporaso et al. (2011) (i.e., 515F and 806R).

The PCR mixture was composed of 65 μl of DNA diluted at 1 ng/μl and 35 μL of mix. Mix was composed of 18 μl of nuclease-free water, 10 μl of 10× buffer, 2 μl of each primer at 20 μM, 2 μl of dNTP at 10 mM, and 1 μl of MolTaq (MolZym, Plaisir, France) at 5 U/μl. DNA template was amplified according to the following thermal conditions: 1 min at 94°C, 28 cycles composed by 30 s at 94°C, 1 min at 56°C, 1 min at 72°C, and a final extension step at 72°C for 10 min. Ten microliters of each product was used to verify amplification by gel electrophoresis on a 1% agarose gels stained with GelRed (Interchim, Montluçon, France).

Paired-end sequencing was performed on a MiSeq System (Illumina, San Diego, CA, United States) with v3 reagents, producing 250 bp reads per end, according to manufacturer’s instructions by the GeT-PlaGe platform (INRA-Toulouse, France). The 16S rDNA reads were demultiplexed by Illumina run software at GeT-PlaGe platform. Reads were then analyzed with the FROGS v3 pipeline (Escudie et al., 2018) in Galaxy environment^1^ as described in Supplementary Material and Methods.

### Eating Behavior Assessment

The behavior tests performed on F344 recipient rats are summarized in Figure 1B and detailed below.

#### Milk Intake

At PND15, milk intake was estimated by the weight-suckle-weight (WSW) method adapted from Sevrin et al. (2017). Pups were weighted (m1, g) and separated from their mother for 2 h to ensure gastric emptying. Before returning to their mother, urine excretion was stimulated by rubbing the urogenital area, and pups were weighted (m2, g) to estimate the hourly metabolic waste weight (m2 − m1)/2. After 1-h suckling, pups were weighted again (m3, g). Milk intake was estimated as the relative bodyweight gain for each pup (m3 − m1) + absolute value (m2 − m1)/2.

#### Liquid Taste Preference (Two-Bottle Choice Test)

Preference for sweet and fat tastes was measured at PND70 using the two-bottle choice test experiment as previously described (Paradis et al., 2017). After a 2-day period of adaptation to the presence of two bottles in their own cage, rats were free to choose between a bottle of tap water (control) and a bottle containing tap water with saccharin (0.01%, *w*/*v*) for sweet taste, or a bottle of tap water with xanthan gum 0.3% (control) and a bottle with corn oil (0.1%, *w*/*v*) and xanthan gum 0.3% for fat taste. Taste solutions provide no or negligible amount of calories to drive caloric satiety response (Paradis et al., 2017). Drink intake was measured daily for 3 days, and bottles were daily inverted to prevent position preference bias. The sweet and fat preference scores were calculated as the ratio between the volume of saccharin or fat solution consumed and the total drink intake in 24 h and then multiplied by 100. A preference for one of the drinks is defined above 50%.

#### Food Motivation

From PND110 to PND125, food motivation was assessed with the straight alley test as described by Pecina et al. (2003) and Wong et al. (2009). This was performed during the active phase, under red light using a 2-m-long black plastic device composed of a starting box (SB) (20 cm × 10 cm × 30 cm), a central alley (160 cm × 10 cm × 30 cm) and a goal box (GB) containing food reward (20 cm × 10 cm × 30 cm). Transparent movable Plexiglas doors separated the SB and the GB from the alley allowing choosing the setting of the GB along the alley. In order to choose the most palatable food reward, animals were habituated to the open-field area then tested in it with several palatable food [milk chocolate (Milka, Mondelez, France), chocolate cereals (Chocapic^®^, Nestlé, Switzerland), ham (Fleury Michon, France), almond paste (Maître Prunille, France), potato crisp (Original Pringles, Kelloggs, United States), and cheese (Leerdammer^®^, Bel, France)]. The number of nose contacts with each food was counted. Males demonstrated a preference for cheese and females for almond paste therefore for the following test, cheese (Leerdammer^®^, Bel, France) was used as food reward for males whereas almond paste (Maître Prunille^®^, France) was used for females.

Before the beginning of the test, a bit of cheese or almond paste was given for 3 days to male and female Fischer offspring, respectively, in their own cage, to extinguish food neophobia. During the training period, animals were fasted overnight (16–17 h) and trained in the afternoon. At D1, rats were placed one by one in the SB and after 30 s adaptation, Plexiglas door was removed and animals were free to go to the GB containing the reward pellet (5 g). Maximal duration of the test was 2 min. During the next training sessions, the distance between the SB door and GB was increased from 30 to 60, 100, 140, and 160 cm (maximum distance). For the test (D10), animals were fed *ad libitum* overnight in order to measure motivation rather than hunger. All trials were video-recorded and position, running, pausing, rearing, and turnaround behaviors, as well as latency to leave the SB, to reach GB and eat reward pellet were measured by a trained “blind” experimenter using BORIS^®^ software (v.7.7.3) (Friard et al., 2016) (ethogram in Supplementary Table 3). An ingestion score was calculated from the latency to ingest the food reward with 1 point removed each 30 s (ingestion between 0 and 30 s = 3 points; between 31 and 60 s = 2 points; 61 and 90 s = 1 points; 91 and 120 s = 0 point). Since straight alley test is based on locomotor activity, a 5-min recorded open-field test was done the day after trial 10 to check locomotor activity or anxiety that showed no differences between rats.

#### Twenty-Four-Hour Eating Pattern

Between PND137 and PND155, eating behavior was analyzed in physiological cages (Phecomb cages, Bioseb, Vitrol, France), as previously described (Coupe et al., 2011; Le Drean et al., 2019). Briefly, rats were allowed to adapt individually to physiological cage during 1 day before data recording. Data were recorded from the beginning of the second day (8:00 a.m.) each 5 s over a 24-h period. Meal was defined as 0.1 g food intake during a minimum of 10 s with an intermeal period of 10 min minimum. Meal parameters extracted from Compulse software (v.1.1.01) (PheCOMP, Panlab, Spain) included latency to eat, number of meals, meal size, and duration of intermeal interval and satiety ratio. A percentage of reliability of the quality data was calculated by the software, and only behavioral items reaching a percentage of reliability >80% have been used.

#### Behavioral Satiety Sequence Analysis

At PND130, the analysis of the behavioral satiety sequence (BSS) was performed as described (Halford et al., 1998) in animal home cage. This was done by videotaped, under red light, animals that received 90 g HED (58V8, TestDiet, Richmond, United States) in their home cage for a 90-min period after an overnight fast. Total food intake was measured, and activities as feeding, grooming, resting were quantified. The test period was divided into eighteen 5-min time bins, which allowed determining meal duration, feeding rate, grooming, and resting time, as well as the time of occurrence of satiety. One female F-Sham and one male F-OP were removed for demonstrating an abnormal lethargy.

#### Behavioral *Z*-Scoring

In order to reduce the intrinsic variability of single tests, we integrated complementary and convergent measures by *z*-normalization along a same behavioral dimension (propensity to eat, preference for more sweet or fat, see Supplementary Table 4), as previously described (Guilloux et al., 2011). This *z*-normalization was calculated according to group and sex.

### Statistical Analysis

All univariate statistical analyses were performed with GraphPad PRISM^®^ software (v.6.01). Because of a low number (*n* < 8) of weaned female OR, we could not check for normality, and because of large differences in variance between groups, we applied non-parametric tests to all datasets, unless otherwise stated. All parameters of growth, food consumption, and eating behavior were analyzed using Kruskal–Wallis analysis of variance and comparison between groups using *post hoc* Dunn’s test. Wilcoxon signed rank test was used to compare group values to a reference value [calorie intake under SD during the first 10 days on HED, no preference–no aversion (50%) during liquid preference test and zero for behavior *z*-scoring].

Linear discriminant analysis effect size (LEfSe) (Segata et al., 2011) was used on the online Galaxy interface (see Text Footnote 1) to investigate bacterial markers that drive differences between groups. Other multivariate statistical analyses were performed under R^®^ (v.3.6.1.) with the FactoMineR^®^ 2.2 Package (Le et al., 2008). Beta diversity was analyzed through clustering of dissimilarity measures (Cao) using the ggdendro^®^ (v.0.1-20) and phyloseq^®^ (v.1.28.0) packages (McMurdie and Holmes, 2013). Relations between eating behavior characteristics and microbiotal features were searched using unfold principal component analysis (UPCA) and by calculation of Spearman correlation coefficients and their level of significance. Using a classical principal component analysis, UPCA helps to link the most relevant behavioral characteristics to the most relevant bacterial abundances, in a simplified subspace of the initial datasets. When these analyses were done on data arising from different ages (i.e., different animals, as, for example, when associating microbiotal features at PND21 and eating behavior characteristics at PND200), the pair fitting was made on the basis of sex and litters belonging.

## Results

### Inocula From OP and OR Dams Differed

**FIGURE 2:**
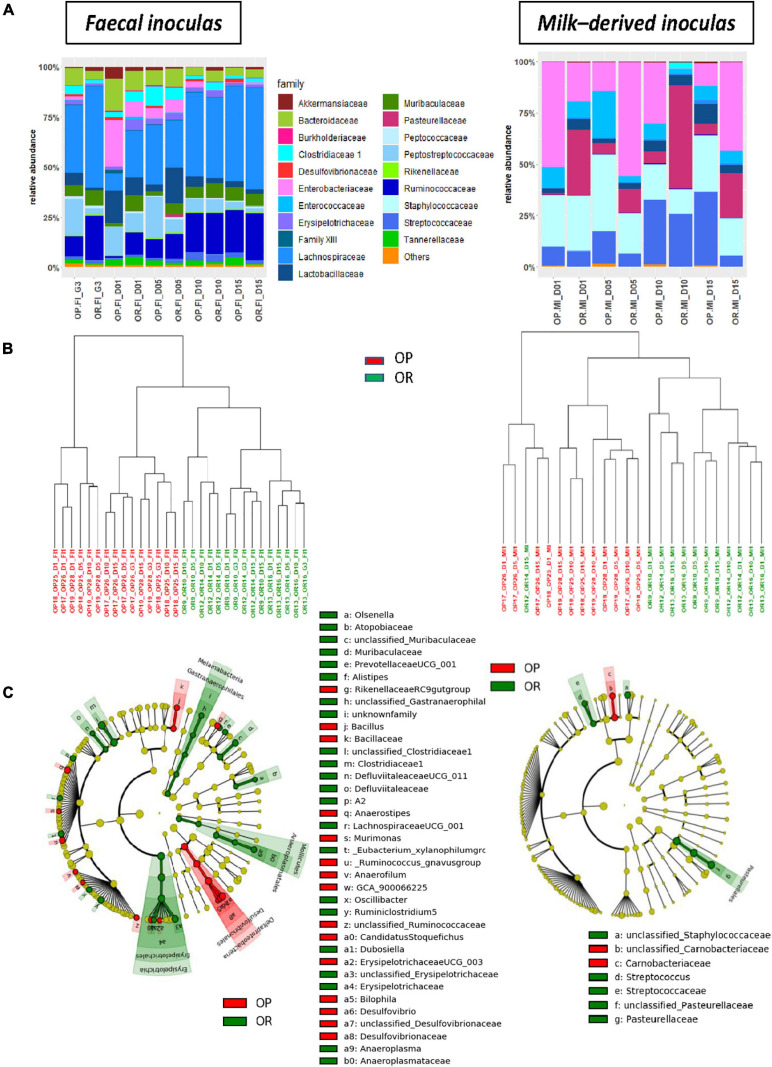
Bacterial composition and structure of inocula prepared from OP and OR dams (*n* = 3 per genetic origin and sampling time). **(A)** Relative abundances of bacterial family and **(B)** Cluster dendrograms of dissimilarities measures (Cao) in inocula according to sampling time (OP-inocula are in red and OR-inocula are in green). **(C)** Taxonomic cladograms obtained from LEfse analysis of OTU abundances, showing as circles (which diameters are proportional to abundances), taxa that significantly (*p* < 0.01) discriminate inocula according to their genetic origins, on concentric rings from domain in the innermost one to genera in the outermost (see Supplementary Figure 1 for LDA values).

As expected, fecal inocula exhibited higher OTU richness than milk-derived ones (Supplementary Table 5), and their bacterial composition were very different (Figure 2A for the family level). Milk-derived inocula predominantly consisted of *Enterobacteriaceae* (ca. 28%), *Staphylococcaceae* (ca. 23%), *Streptococcaceae* (ca. 17%), and *Pasteurellaceae* (ca. 17%) while fecal inocula composition was dominated by *Lachnospiraceae* (ca. 35%) and *Ruminococcaceae* (ca. 15%).

Within each inoculum sampling site and taking into account all sampling times, shared OTUs constituted the major fraction (79.2 and 48.7% for feces- and milk-derived inocula, respectively), but differences according to the genetic background were observed. These were found to be insignificant on alpha-diversity indexes (Supplementary Table 5), but clear-cut separations were evidenced by clustering of dissimilarity measures (Cao) between samples (Figure 2B). We also identified various taxa with significantly differential abundances according to the genetic background using LEfSe analysis (Figure 2C). These were numerous for fecal inocula with the highest LDA scores being observed for members of *Clostridiaceae 1*, *Tannerellaceae*, and *Erysipelotrichaceae* (more prevalent in OR), as well as members of *Desulfovibrionaceae*, *Parabacteroides*, or *Anaerostipes* (more prevalent in OP, Supplementary Figure 1A). Conversely, milk-derived inocula from OP were discriminated from those originating from OR by only four genera and one species: *Streptococcus* sp. and two unclassified genera affiliated to *Pasteurellaceae* or *Staphylococcaceae* were more prevalent in OR while one unclassified genus among *Carnobacteriaceae* and one unclassified species from *Staphylococcus* sp. were more prevalent in OR (Figure 2C and Supplementary Figure 1B).

Characterization of vaginal inocula did not succeed due to extremely low concentrations of bacterial DNA (1.0 ± 0.4 ng/μl), and their composition based on 16S sequencing was equivalent to that observed in the negative controls (data not shown).

### Inocula Transfer Induced Transient Changes in Gut Microbiota of Recipients

**FIGURE 3:**
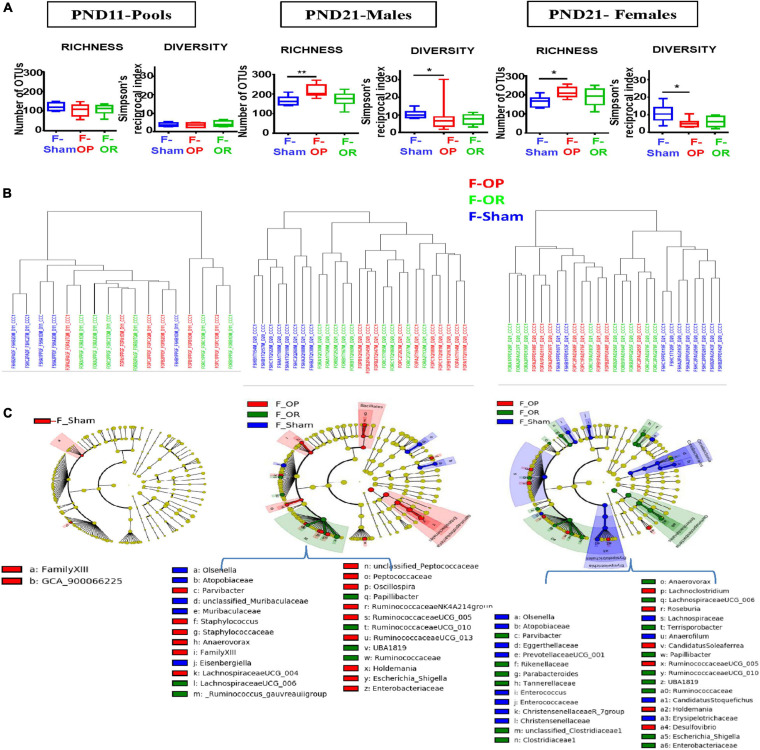
Structure and composition of cecocolonic microbiotas of F344 recipients before weaning (*n* = 5 to 10 samples per group and per sex). **(A)** Observed richness and diversity. **(B)** Cluster dendrograms of dissimilarity measures (Cao) between individual samples (F-Sham are in blue, F-OP in red, and F-OR in green). **(C)** Taxonomic cladograms obtained from LEfse analysis of OTU abundances, showing as circles (which diameters are proportional to abundances), taxa that significantly (*p* < 0.05) discriminate samples according to the experimental group, on concentric rings from phyla in the innermost one to species in the outermost, see specific color legend for each sampling time (see Supplementary Figure 3 for LDA values).

Inocula transfers modified the composition of intestinal microbiota in F344 recipients before weaning (Figures 3A–C) without significantly impacting the absolute amounts of bacteria (Supplementary Table 6). Microbiotal changes were mainly observed at PND21, i.e., 6 days after the last transfers. Indeed, at PND11, cecocolonic bacterial communities of F-sham, F-OP, F-OR, and F344 recipients did not significantly differ in terms of alpha- and beta-diversities nor dominant families (Figures 3A–C). However, following LEfSe analysis (Figure 3C), gut microbiota from both the F-OP and F-OR groups stood out significantly from the F-Sham group by their contents in bacteria of *Family XIII* (LDA score = 2.47) and in an unknown species affiliated to the GCA-900066225 group in *Ruminococcaceae* (LDA score = 2.06) (Supplementary Figure 2).

Conversely, at PND21, observed richness was increased while alpha diversity, as estimated by Simpson’s reciprocal index (i.e., 1/D), was decreased in females and males from the F-OP group as compared with their F-Sham counterparts (Figure 3A). In addition, in a very obvious way for males and to a lesser extent for females, clear differences between intestinal microbial communities were demonstrated by hierarchical clustering of dissimilarity values (Cao) between microbiotas from the F-OP and F-OR groups—which did not separate from each other—and those from the F-Sham group (Figure 3B). Accordingly, many taxa were identified by the LEfSe analysis as characteristic of both the F-OP and F-OR groups, when compared with the F-Sham group (16 for females and five for males, Figure 3C). However, this analysis also allowed the identification of taxa specific of either the F-OP or F-OR group (six and 19 for females and males from the F-OP group and 18 and six for females and males from the F-OR group). These taxa belong to both ultradominant and subdominant families (e.g., *Lachnospiraceae* and *Atopobiaceae* in the case of the F-Sham females) (Supplementary Figure 3A). Depending on the group, the nature of these taxa drastically differed or not for each sex. For F-OR, only an unclassified species belonging to the *Holdemania* sp. constituted a marker found in both males and females. Conversely, in the case of F-OP, the five taxa identified as characteristic in males were also found in females with LDA scores of the same order of magnitude (see Supplementary Figures 3A,B).

After weaning, in growing and adult animals, analyses led to heterogeneous results suggesting that microbiotal changes, if any, were of small magnitude. Using the LEfSe analysis, a few taxa were identified as characteristic of the different groups, at most sampling times for both sexes, but no overall picture emerged from the time course of their numbers as the animals aged (Supplementary Figures 4A–D). Within a same group, most of these bacterial markers differed according to sex or age. The few exceptions were (i) an unknown species from *Candidatus Soleaferrea* sp. which was associated to the F-OP group at both PND130 and PND200 in males and (ii) the genera *Ruminiclostridium* and *Enterorhabdus* which were associated to the F-OR group in both males and females. In addition to a larger number of associated taxa, the F-OR group was also distinguished by significant differences in observed richness, which was increased in females from the F-OR group at PND60 (257 [226–282] as median and [range]) and PND130 (299 [281–310] as compared with their counterparts from the F-Sham group (224 [184–244] and 272 [241–285], respectively). Conversely, experimental groups could not be distinguished anymore by the beta-diversities of the microbiotal communities (Supplementary Figure 5).

**FIGURE 4:**
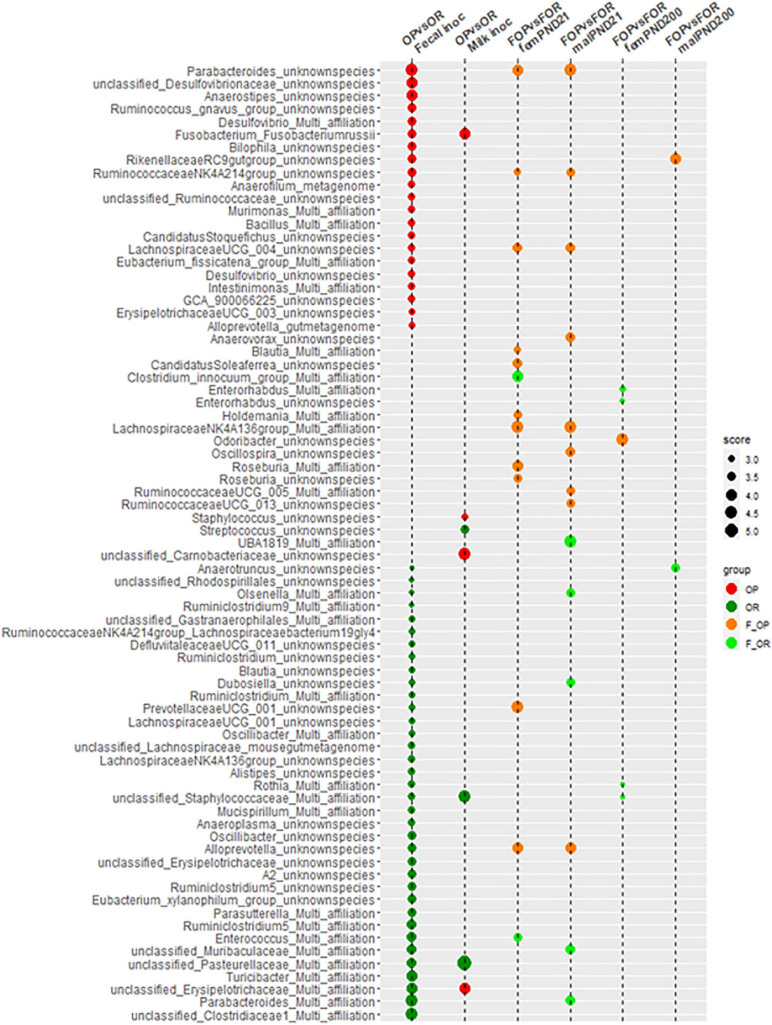
Compilation, at the bacterial species level, of the LDA scores obtained during comparisons carried out between inocula and between F344 recipients.

When compiling all the bacterial species identified as discriminative of the different types of microbiota (Figure 4), it appears that very few of those identified from the inocula were retrieved in the F344 recipients. Therefore, the modification of the F344 recipients’ microbiota by transfer did not identically replicate the transferred inocula.

### Neonatal Transfer of Maternal Microbiota Had Little Effect on Early Growth but Impacted Feeding Behavior

#### Pup Growth Before Weaning

**FIGURE 5:**
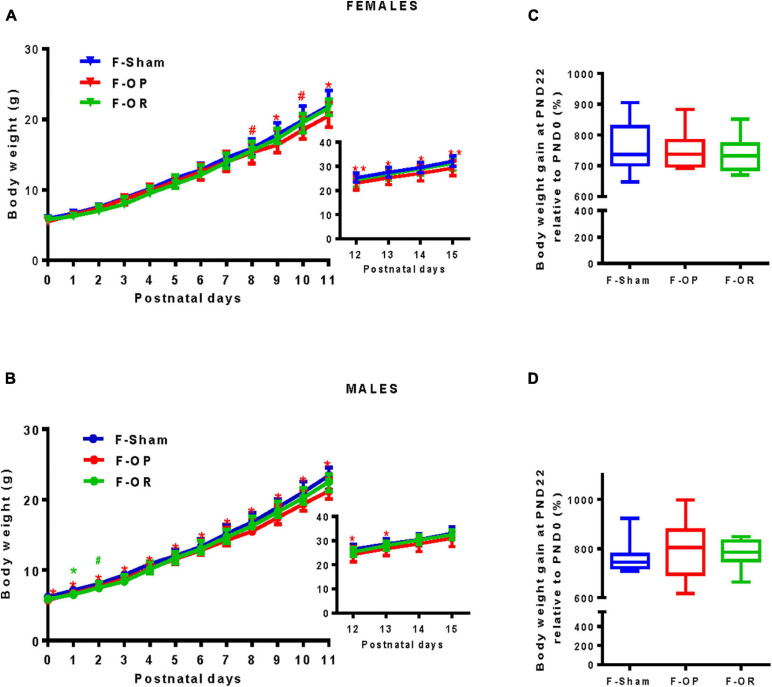
Pups’ growth during the lactation period. Bodyweight of female **(A)** and male **(B)**, from birth to PND11 (females *n* = 22–25, males *n* = 23–26) and in inserts from PND12 to PND15 (females *n* = 16–19, males *n* = 17–20), data are medians and interquartile range, *p < 0.05, **p < 0.01, ^#^*p* < 0.1, Kruskal–Wallis and *post hoc* Dunn’s test at each PND. “*” and “**” indicate differences (“^#^” tendency) between F-OP and F-Sham; “*” indicates differences (“^#^” tendency) between F-OR and F-Sham. **(C,D)** Relative bodyweight (%) obtained by dividing body weight gain at PND22 by body weight at birth of females (**C**, *n* = 7–9) and males (**D**, *n* = 9–11).

Pup bodyweight was monitored daily from birth until PND15 (Figures 5A,B). Except for one transient difference at PND1 and a trend at PND2, no significant difference was observed between the F-OR, F-Sham, and F-OP groups. Both female (Figure 5A) and male pups (Figure 5B) from the F-OP group weighed less than their counterparts from the F-Sham group. This difference reached statistical significance from PND9 until PND15 for females and through the entire period for males. Nevertheless, no significant difference was observed for body weight gain measured at PND22 (only on fully weaned pups) (Figures 5C,D).

#### Milk Intake

**FIGURE 6:**
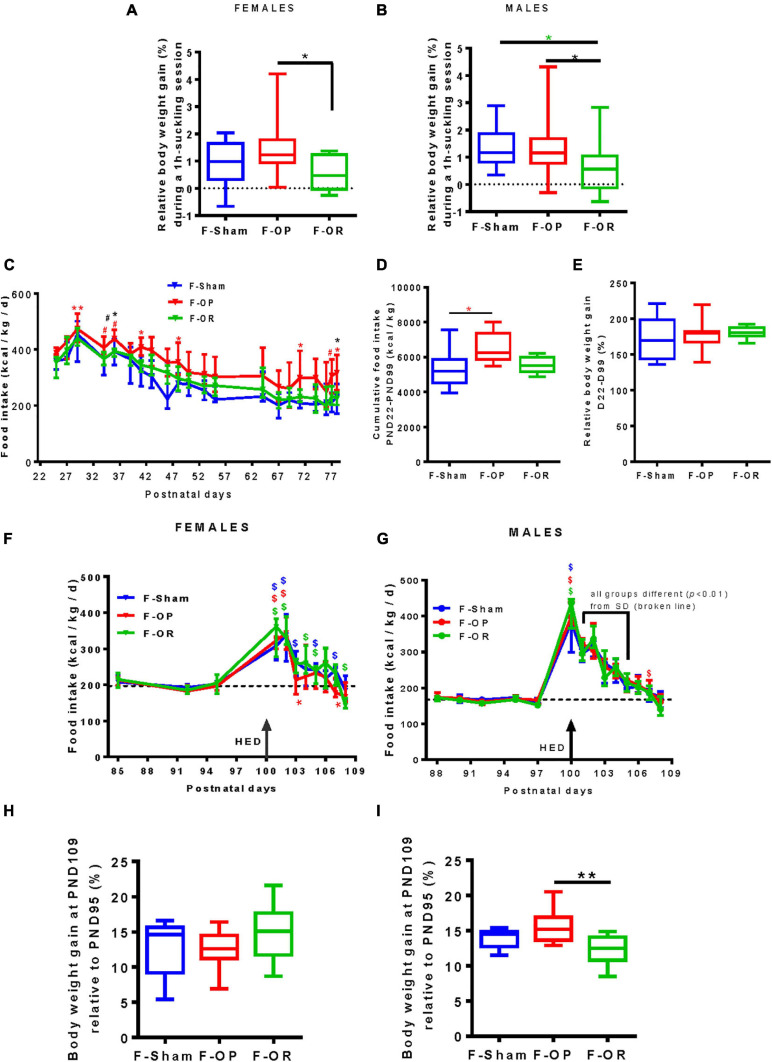
Food intake at different periods of life. Hourly milk intake of female **(A)** (*n* = 16–19) and **(B)** male pups (*n* = 17–20) at PND15 estimated by the body weight gain after suckling divided by initial body weight. Daily **(C)** and cumulative **(D)** food intake and **(E)** relative weight gain on SD after weaning in females (*n* = 7–9). **(F,G)** Daily caloric intake and **(H,I)** relative weight gain during the first 10 days of HED (PND100–109) in females (left, *n* = 7–8) and males (right, *n* = 9–11). Panels **(A,B,D,E,H,I)** data are medians with range (min–max), *p < 0.05, **p < 0.01, Kruskal–Wallis and *post hoc* Dunn’s tests. **(C,F,G)** Data are medians and interquartile range, *p < 0.05, **p < 0.01, ^#^0.05 < *p* < 0.06; “*” indicates differences (“^#^” tendency) between F-OP and F-Sham; “*” indicates differences (“^#^” tendency) between F-OP and F-OR. **(F,G)** ^$^, ^$^, ^$^*p* < 0.05 indicate difference from basal SD consumption (dotted line, median of the three groups) in each group, Wilcoxon signed rank test.

Hourly milk intake (as estimated by relative weight gain after 1-h suckling) at PND15 was significantly lower for both male and female F-OR than F-OP pups and lower for F-OR males as compared with F-Sham (Figures 6A,B).

#### Pups’ Growth After Weaning

From PND25 to PND78, energy intake from SD was higher for F-OP females than F-Sham females (Figures 6C,D) but without any effect on weight gain (Figure 6E). During the same period, no significant difference between groups of males was observed (data not shown).

Rats were then switched to HED from PND100 to the end of the experiment (PND200), and food consumption was recorded daily from PND99 to PND109. Their energy intake rose to stabilize later and differently between females and males. For females, no difference in energy intake was detected between groups during the first 10 days of HED (Figure 6F) nor in body weight gain (Figure 6H). F-OP females adjusted their food intake in 3 days, when F-Sham and F-OR adjusted their food intake in 7 days (Figure 6F). Concerning males, despite no difference in caloric ingestion between groups during the first 10 days of HED (Figure 6G), F-OR gained significantly less weight than F-OP rats (Figure 6I). F-Sham and F-OR males adjusted their food intake in 7 days, when F-OP males needed 8 days (Figure 6G).

During the behavioral tests from PND110 to PND155, no significant difference in body weight, body weight gain, or food intake was noticed between groups among female and male F344 recipients.

#### Liquid Taste Preference (Two-Bottle Choice Test)

**FIGURE 7:**
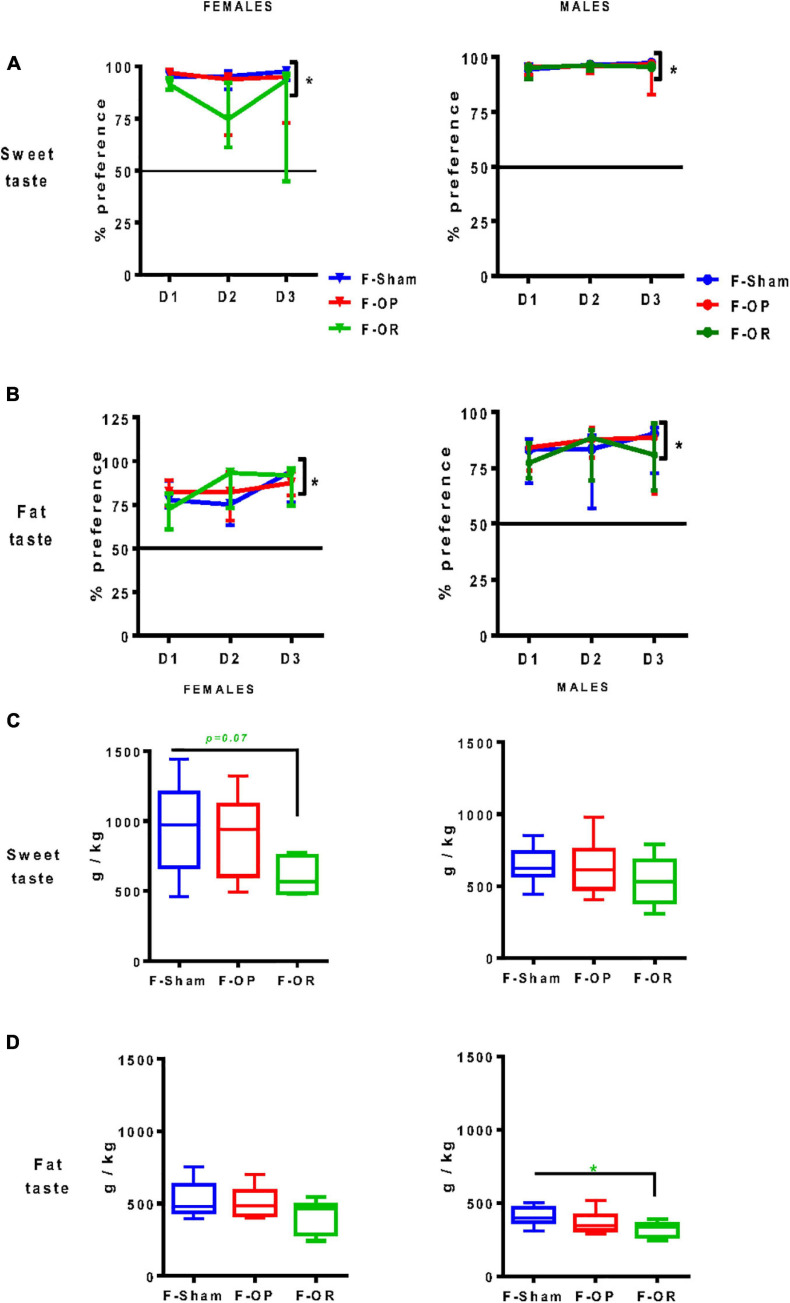
Liquid taste preference. Preference for sweet **(A)** and fat **(B)** tastes and total intake of liquids drunk during the 3-day test for sweet **(C)** and fat **(D)** tastes in females (left, *n* = 7–9) and males (right, *n* = 8–11). Data are expressed as medians and interquartile range. **(A,B)** *p < 0.05, indicates difference from 50%, Wilcoxon signed rank test. Differences between groups were characterized by Kruskal–Wallis and *post hoc* Dunn’s at each day of the test. **(C,D)** *p < 0.05, Kruskal–Wallis and *post hoc* Dunn’s test. Animals were 75–85-day old and fed SD.

From PND75 to PND85, taste preference was measured using the *two*-*bottle choice test*. All animals significantly preferred sweet- and fat-tasting solutions, expressed as a percentage of consumption of taste solution above 50%, and no difference was observed between groups (Figures 7A,B). However, the total intake of sweet solution during the 3-day test tended to be lower in F-OR females (Figure 7C) and the total intake of fat solution was significantly lower in F-OR males (Figure 7D).

#### Food Motivation

**FIGURE 8:**
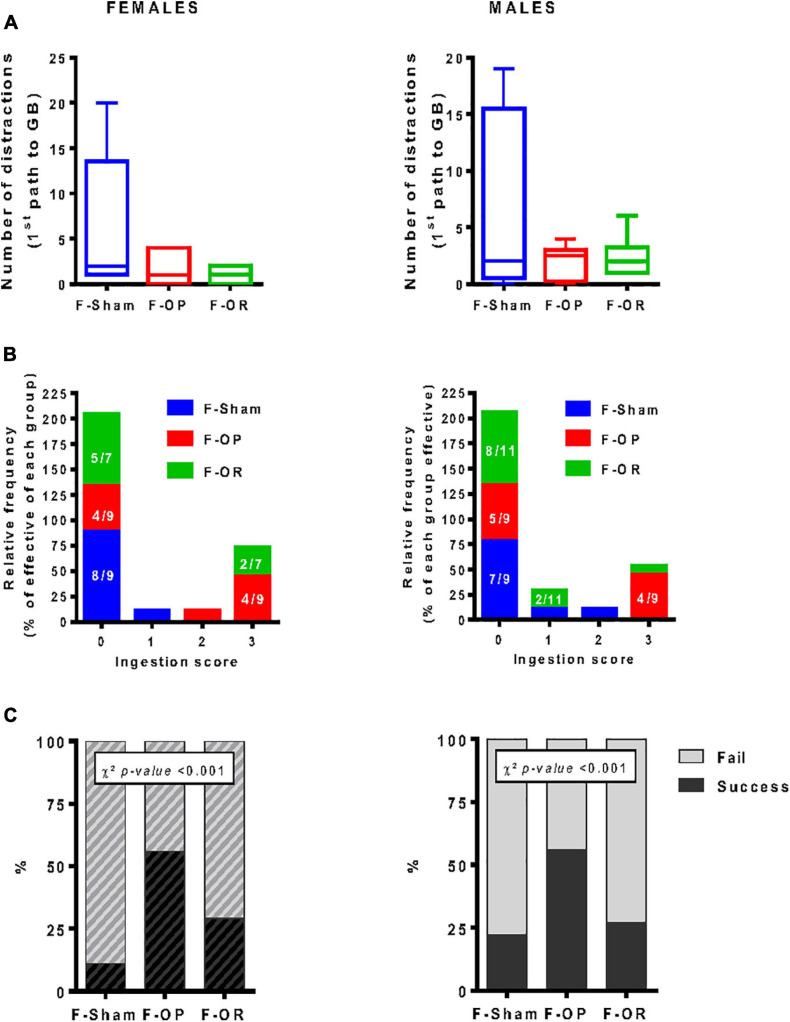
Food motivation (straight alley test). **(A)** Number of distractions (stops and rearing) during the first walk to food reward, data are medians and min–max, Kruskal–Wallis test. **(B)** ingestion score (velocity to eat the food reward, scale 0 (lower score) to 3 (higher score), data are number values for each score (frequency). **(C)** Percentage of test success (eat reward)/failure (not eat reward), uniformity χ^2^ test. **(A–C)** Left: females (*n* = 7–9), right: males (*n* = 9–11). Animals were 110–125-day old and fed HED.

From PND110 to PND125, food motivation was assessed by the *straight alley test*. On the test day, both F-OP and F-OR females and males seemed to be more focused than the F-Sham groups on their way to reach the GB, with less stops and rearing, but this effect was not significant (Figures 8A,B). Both male and female F-OP showed a higher frequency of eating the reward (score 3) compared with F-Sham and, to a lesser extent, F-OR (Figure 8B). The greater performance in the F-OP groups was revealed by a Chi^2^ test showing significantly more success in eating the reward, for females and males (Figure 8C). These differences did not depend on the overall activity (locomotion or stops) during the first path to the palatable reward (GB) (data not shown) or a state of anxiety that was evaluated 1 day after the straight alley test in the open-field test (data not shown).

#### Twenty-Four-Hour Eating Pattern

**FIGURE 9:**
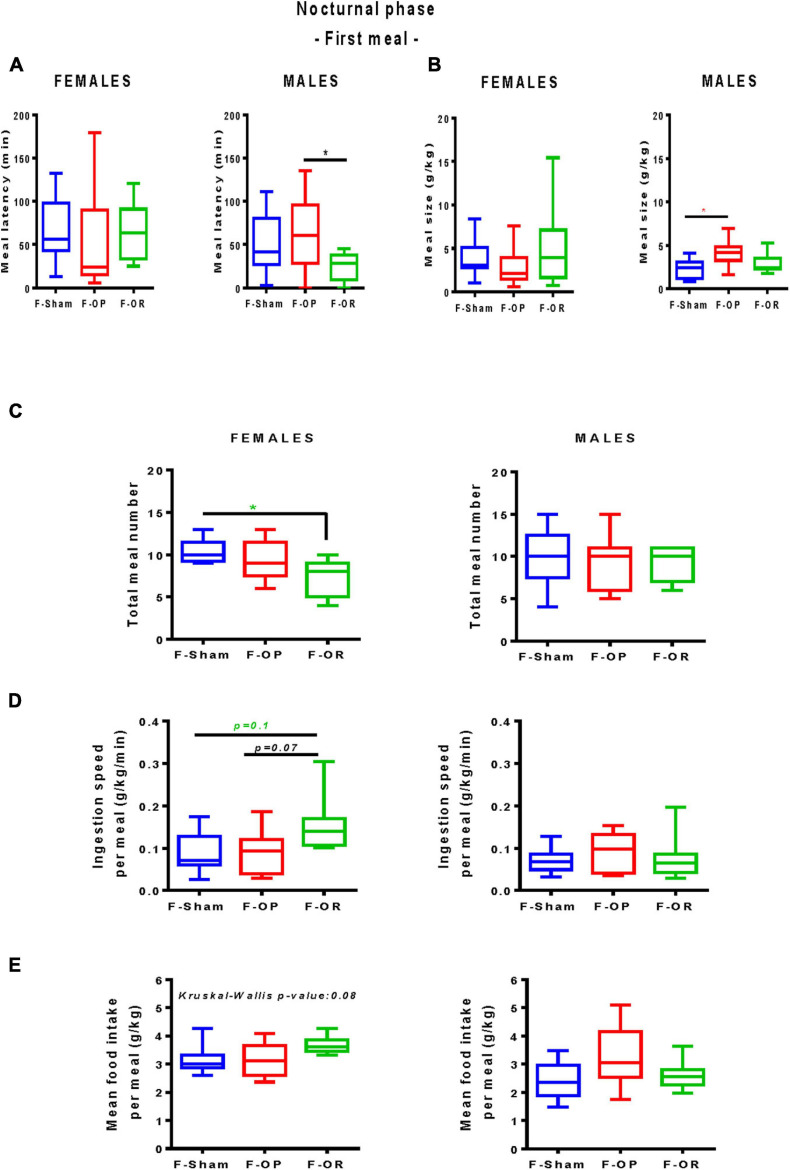
Meal pattern. **(A)** Latency to eat and **(B)** meal size during the first nocturnal meal. **(C)** Total meal number. **(D)** Ingestion speed per meal and **(E)** mean food intake per meal during the nocturnal phase (12 h). Left: females (*n* = 7–9), right: males (*n* = 9–11). Data are medians and min–max values; *p < 0.05 indicate difference between groups, Kruskal–Wallis and *post hoc* Dunn’s test. Animals were 137–155-day old and fed HED.

From PND137 to PND155, meal pattern was characterized in detail over a 24-h period. No significant difference in total food intake was noticed between groups. During the 12-h diurnal phase, F344 recipient rats consumed a negligible amount of food that did not differ between groups (data not shown); we therefore focused on the nocturnal phase. No difference was revealed concerning the first nocturnal meal of the females (Figures 9A,B). Male F-OR consumed their first nocturnal meal significantly quicker as compared with male F-OP (Figure 9A), and the meal was bigger for male F-OP than male F-Sham (Figure 9B). During the 12-h dark period, in female F-OR, the number of meals was smaller as compared with F-Sham (Figure 9C), with a trend toward a higher ingestion speed per meal (Figure 9D), but mean food intake per meal did not significantly differ (Figure 9E).

#### Behavioral Satiety Sequence

**FIGURE 10:**
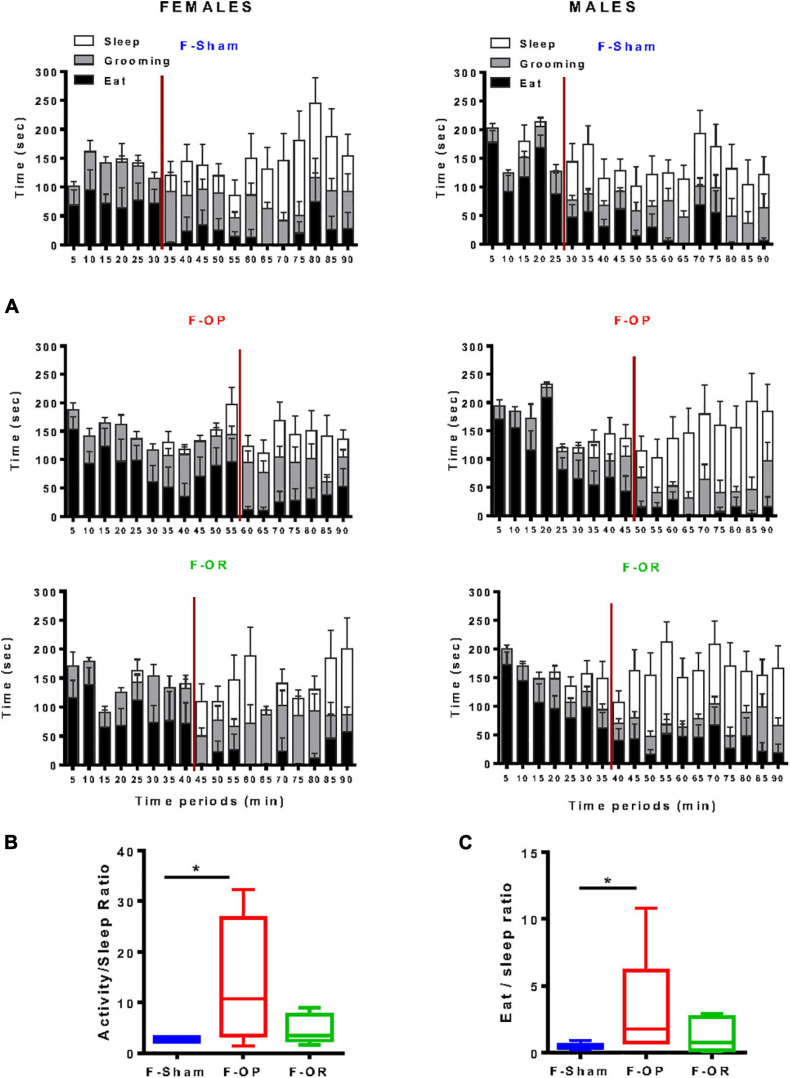
Behavioral satiety sequence. **(A)** Blundell representations of behavioral satiety sequences for the three groups of females (left, *n* = 7–9) and males (right, *n* = 8–11), data are means ± SEM, satiety point is represented by the red vertical line and correspond to latency before behavioral switch between eat and sleep. **(B)** Activity-to-sleep ratio and **(C)** eat-to-sleep ratio in females (*n* = 7–9); data are medians and min–max values, *p < 0.05 indicates difference between groups, Kruskal–Wallis and *post hoc* Dunn’s test. Animals were 130-day old and fed HED.

Behavioral satiety sequence was monitored over a 90-min period at PND130. Females and males from the F-Sham group exhibited a typical BSS characterized by an initial eating period of approximately 30 min followed by grooming, then sleeping (Figure 10A). In females, the satiety state occurred after 40 and 55 min for F-OR and F-OP, respectively. The longer eating period featured by F-OP than F-Sham females resulted in a significant increase in the “activity/sleep” and “eat/sleep” ratios (Figures 10B,C).

In males of the F-Sham group, the sleeping period started 25 min postrefeeding (Figure 10A), around 35 min for the F-OR group and 45 min for the F-OP, but due to the lack of clearcut difference between behaviors, no significant difference was observed in activity/sleep and eat/sleep ratios between male groups (data not shown).

#### Integrated Behavioral *Z*-Score

**FIGURE 11:**
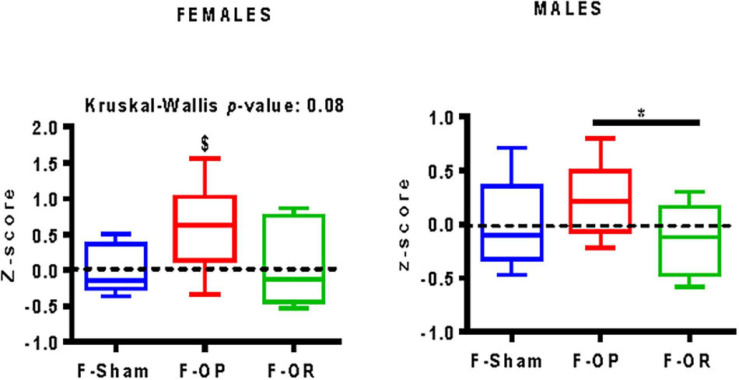
Integrative calculated behavioral *z*-score of females (left, *n* = 7–9) and males (right, *n* = 9–11), data are median and minimum–maximum values; *p < 0.05 indicates difference between groups, Kruskal–Wallis and *post hoc* Dunn’s test, ^$^*p* < 0.05 indicates difference from the reference score value (zero), Wilcoxon signed rank test.

For F-OP females, the integrated behavioral *z*-score was significantly greater than zero, which means for this group an increased risk of eating behavior which favors higher food consumption, a preference for fat or sugar, and higher motivation for food (Figure 11). For males of the F-OP group, the integrated behavioral *z*-score was significantly higher than that of their F-OR counterparts, conferring to the former a riskier eating behavior.

### Behavioral Characteristics of Adults Were Associated With Both Juvenile and Adult Microbiotal Traits

To determine whether the feeding behavior in adult recipient rats could be associated with specific characteristics of the microbiota, we performed an unfold PCA analysis which is a simple and straightforward method of analysis to investigate the relationships between two datasets after pair fitting made on the basis of sex and litters belonging when necessary. As a few data are lacking for some animals regarding behavioral tests, the analysis could not be applied to all individuals.

When considering microbiotal data at PND21, for males and females, no specific relationship could be detected between eating behavior characteristics and bacterial family abundances, which both contribute mostly to the first two components (Supplementary Figure 6A). Conversely, for OTU abundances (Supplementary Figure 6B), both “total cumulated SD consumption” and “SD efficiency” appeared related to the abundance of one specific member of *Lactobacillus* sp. (cluster 470) and of one OTU affiliated to *Ruminococcaceae* NK4A214 group (cluster 251) in females while the “average duration of sleep” was related to the abundance of one unclassified *Lachnospiraceae* (cluster 93) in males. All these associations were observed while UCPA allowed a good separation between individuals from the Sham groups and those from the FOP and FOR groups (data not shown) and therefore may have been induced by treatments.

Regarding adult microbiota abundances, associations were only found for females for which both “blundell_eat.to.sleep_min” and “HED efficiency during the first week” were aggregated with abundances of three specific OTUs, belonging to unclassified *Lachnospiraceae* (cluster 134), *Oscillobacter* sp. (cluster 343) and *Ruminiclostridium 6* (cluster 449), respectively (Supplementary Figure 7).

**FIGURE 12:**
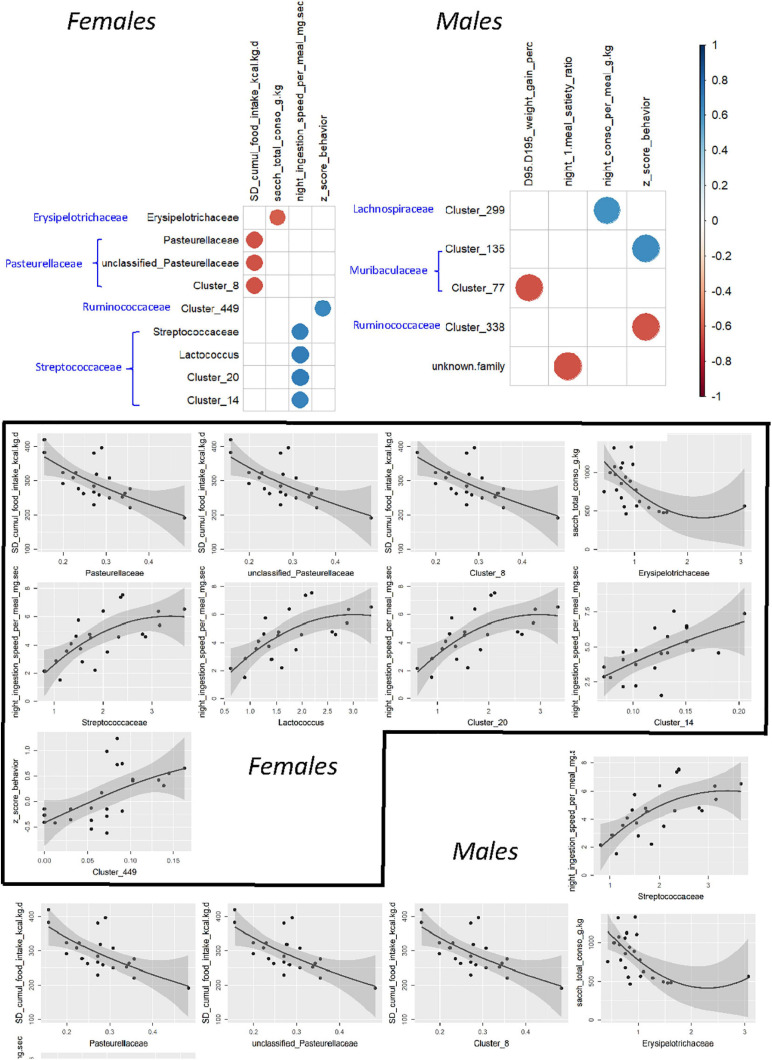
Correlograms showing significant (α < 0.01) Spearman rank correlations among selected components of eating behavior and the relative abundances of some bacterial taxa in F344 recipients and corresponding scatter plots, using individual data from PND200 female and male animals. In all correlograms, positive correlations are displayed in blue and negative correlations in red as scaled on the right. The black line and associated gray zone represent a LOESS smoother and the 95% confidence interval, respectively.

To further investigate associations between microbiotal and behavioral characteristics, a search for Spearman correlation between individual variables was carried out after selecting behavioral variables representative of the functions affected by treatment. Interestingly, significant correlations were found not only for bacterial abundances measured at adulthood, i.e., at the same stage of life as behavior testing but also for those determined before weaning (Figure 12). In both cases, the identified taxa belonged to a large range of bacterial families, but this was exacerbated when using preweaning microbiotal data. None of the correlated couples that were identified using the PND21 data was recovered when using 16S data from adults. The sole exception was a correlation between members of *Ruminococcaceae* and the integrated behavioral *z*-score in females; however, the precise nature of these members differed, while the direction of the correlation was inverted.

For females, the strongest associations (Supplementary Figure 8A) corresponded to (i) an inverse relationship between the “satiety ratio after the first night meal” and the abundance of the *Clostridium innocuum* group or one of its constituent clusters (cluster 102) at PND21, (ii) an inverse relationship between the “cumulated consumption of SD” and the abundance of the family *Pasteurellaceae* or two of its components (an unclassified genus and cluster 8), and (iii) a positive relationship between “the rate of ingestion per night meal” and the abundance of the *Streptococcaceae* family or two of its components (*Lactococcus* sp. and cluster 20) in adulthood. For males, there were three inverse relationships, the first between “body weight gain under HED” and the abundance, at PND21, of cluster 77, affiliated to an unidentified genus of *Muribaculaceae*, the second between “HED intake during the first week” and the abundance of cluster 2, affiliated to *Lactobacillus* sp. and the third between the satiety ratio after the first night meal and the abundance of *Bacteroides* sp., at adulthood (Supplementary Figure 8B).

## Discussion

To the best of our knowledge, the current report is the first to demonstrate that the transfer of maternal microbiota from mother to child can program the eating behavior of the offspring in a rodent model of obesity.

We chose to perform a vertical transfer of microbiota of dams that were genetically predisposed to obesity or not, both fed a high-energy diet, to newborn pups born from a control strain, unchallenged Fisher dams, so as to get rid of the genetic determinants of offspring obesity and to minimize confounding factors such as metabolic and the low-grade inflammation due to both obesity *per se* and the high-energy diet during gestation and lactation, in an effort to explore the sole impact of the transfer of different microbiota *per se* on feeding behavior phenotype. Our second objective was to identify whether the differences in the composition of early microbiota were long lasting and were associated with specific characteristics of feeding behavior.

### The Inocula Did Exhibit Different Bacterial Composition but Exerted a Transient Impact on Recipients’ Intestinal Microbiota

The underlying assumption for our experimental design was that the microbiota obtained from several sites differed between OP and OR dams. Scattered studies conducted in either standard Sprague-Dawley or Wistar male rats with differential responsiveness to high-energy diets, or in the Sprague-Dawley OP and OR, which were initially selected (Levin et al., 1997) to respond differently to high-fat diets, support such assumption. Differences in the composition of intestinal or fecal microbiota were reported (de La Serre et al., 2010; Cluny et al., 2015), and Obanda et al. (2018) described a difference in the metabolic capacity of the microbiota, which has since also been associated with changes in the cecal abundance of some specific strains (Obanda et al., 2020).

Despite the known impact of gender on microbiota composition (e.g., Peng et al., 2020), the sequencing of 16S DNA fragments that we carried out revealed significant differences in bacterial composition between inocula prepared from feces or milk depending on the donors’ phenotype. Some of the differences observed concerned bacterial populations already associated in the literature with overweight or metabolic syndrome. For example, our LEfSe analysis indicated that OR inocula were typified by *Clostridiaceae 1* which were previously reported to be more abundant in the lean than overweight children (Golloso-Gubat et al., 2020) and which includes *Clostridium butyricum*, a more abundant species in OR than in OP male rats in the studies of Obanda et al. (2020). Similarly, we identified *Desulfovibrionaceae* as a hallmark of fecal inoculum from OP dams, which matched previous reports associating this family with obesity (Petersen et al., 2019) or dyslipidemia (Marungruang et al., 2018).

However, it probably is simplistic to expect similarities between studies regarding associations between bacterial populations and pathophysiological states when bacterial composition is obtained by sequencing 16S DNA fragment: because of the risks of discordant identifications when using different databases [or different updated versions (Edgar, 2018)] and because of the inadequacy of 16S DNA fragments sequencing to provide appropriate taxonomic resolution, namely at the species or strain levels (Johnson et al., 2019). Indeed, higher taxonomic levels may include both positively correlated members and negatively correlated ones for a given physiological situation. Such a case is observed for the genus *Parabacteroides* in our study. Among the two OTUs that make it up (one identified as an unknown species, the other one associated with a multiplicity of identities), one was recognized as typical of OR fecal inoculum whereas the other one as typical of OP fecal inoculum. In this case, the genus level, and even less the family one, is obviously not appropriate to draw any conclusion, and identification of bacterial biomarkers would require a more detailed characterization of the bacterial composition of samples, e.g., after sequencing of the entire 16S DNA or after shotgun metagenome analysis.

Nevertheless, 16S DNA fragments sequencing is perfectly suited to establish the existence of differences in bacterial compositions, and those we observed between OP and OR inocula validated the animal model we had chosen to study the long-term consequences of vertical mother–neonate transfers of maternal microbiota.

Daily transfers from delivery to PND15 of these inocula did induce changes of cecocolonic microbiota in F344 recipient pups, but such impact was clearly detectable only on PND21. This observation raises two issues. First, the finding of a clear impact 6 days after the end of the inocula transfer while no difference was observed at PND11, i.e., during the exact period when transfers were applied on a daily basis, is surprising at first glance. Since the total amount of bacteria transferred remained constant throughout the transfer period (cf. Supplementary Table 1), it rather likely reflects the delay necessary for transferred bacteria to colonize the gut of the recipient pups, including the time required to produce a favorable environment particularly in terms of redox potential since unlike the first natural colonizers, the bacteria transferred were predominantly anaerobes from fecal inoculum (Pesti, 1979). Second, the difference in composition induced by transfers in recipient pups had vanished by the time they reached adulthood. Based on the longitudinal follow-up we performed, using feces collected at PND60 and PND130 (Supplementary Figures 4A–D), the microbiotal differences between transferred and control animals were minimal as early as PND60. This fading overtime was already observed in our earlier studies using neonatal prebiotic supplementations to induce early microbiological changes (i.e., prior to full weaning) (Morel et al., 2015; Le Drean et al., 2019). Our manipulations of the microbiota composition might in fact have been applied too early as they were stopped before weaning, a time when intestinal microbiota is described as unstable (Schloss et al., 2012) as a result of the change in the nature of the food consumed (see Laforest-Lapointe and Arrieta, 2017 for a review). This instability also stems from the intestinal maturation process which continues at the same time and affects both the immune and epithelial components of the colon, both of which being involved in the control of bacteria engraftment (Biol-N’garagba and Louisot, 2003; Zhang et al., 2015).

Nevertheless, although they had no long-lasting impact on the composition of the microbiota, the neonatal transfers we performed did affect recipients’ physiology.

### Feeding Behavior Phenotype of Fisher Recipients Varied According to OP or OR Microbiota Transfers

The multiple tests used in the present study were intended to reveal differences that would reflect traits of eating behavior predisposing transferred pups to obesity: two tests to measure reward-seeking behavior, the SD to HED switch to reveal the ability to regulate the intake of a high-energy food, the measure of the occurrence of satiety to inform about the postprandial state, and the 24 h feeding pattern to inform about night and day eating characteristics.

Significant behavioral differences were observed between groups depending on each test and according to sex. Because eating behavior is complex, we chose to use an integrated behavioral *z*-score that consolidates most relevant items of the various tests. This composite behavioral *z*-score indicates how many standard deviations (*s*) an observation (*X*) is above or below the mean of a control group (*m*), i.e., *Z* = (*X* − *m*)/*s* as previously used to define anxiety- and depressive-like state in mice by the use of complementary tests (Guilloux et al., 2011; Mir et al., 2020). Regarding the behavioral items used to calculate this score, a value significantly above zero is in favor of a higher risk to develop an over-eating disorder predisposing to obesity and a value significantly below zero is associated with a normal-eating behavior potentially protecting against obesity. The integrated behavioral *z*-score of females F-OP showed a risk of eating behavior which includes higher food consumption and higher reward seeking behavior. For males of the F-OP group, the integrated behavioral *z*-score was significantly higher than that of F-OR counterparts, conferring to the former a riskier eating behavior.

In the sweet and fat preference tests, all groups demonstrated a strong preference for saccharine and corn oil, and therefore the test did not discriminate between groups. However, male and female F-OR had a lower total intake of tasty liquids after 3 days of presentation. So far, little is known on the relationship between taste perception, food preferences, and microbiota, but a few studies suggest that rodent strains created through selective pressure based on taste, the Occidental low- and high-saccharin–consuming rats, outbred on the basis of saccharin intake, harbor different microbial communities (Lyte et al., 2016). Whether the microbiota is causally related to the taste phenotype remains to be determined. The lower total intake of tasty or fatty liquid may reflect the onset of a satiety signal, not dependent on calories, which appeared faster in the F-OR rats and that may be linked with oral or intestinal microbiota composition. Interestingly in gastric bypass models of rats, a similar decrease in voluntary consumption of tasty solutions is reported (Mathes et al., 2015). This result underlines the importance of signals of intestinal origin in the regulation of the consumption of tasty foods, but the involvement of the microbiota remains to be established. In our study, the hedonic component of sugar/fat taste was not altered but postoral signals may differ between the groups to modulate potent negative consequence of an excess intake of these palatable foods (e.g., visceral pain).

In our study, the motivation test revealed a striking finding: Fisher rats that had been transferred with microbiota, regardless of the donor, performed better in the straight alley tests than F-Sham during the 10-day trials (data not shown) and during the final test, and in addition, F-OP performed better than F-OR in the final test, testifying a higher motivation to consume the food for the first group. It has been recently documented that change in gut microbe diversity and richness influence serotoninergic, GABAergic, noradrenergic, and dopaminergic neurotransmission, the latter being the key modulator of decision making, motivation, reward, and memory (Gonzalez-Arancibia et al., 2019). There is some evidence that microbiota-gut-brain axis is the key to the pathophysiology of several neuropsychiatric disorders involving dopaminergic neurotransmission, and many transient and persistent inhabitants of the gut, including *Escherichia coli* or *Enterococcus faecium* have been shown to manufacture DA (Lyte and Lyte, 2019) though availability of such DA to the brain is debated.

Satiety refers to the result of satiation that ends an eating sequence and is a transitional period separating meals. Meal size is controlled by vagal afferent nerve which integrates satiety signals from the enteroendocrine cells to the NTS in the brain stem (Berthoud, 2006). Vagal terminals end in the lamina propria so that they can sense bacterial metabolites or byproducts to modulate food intake, as illustrated by LPS-induced hyperphagia which is probably mediated by vagal TLR4 receptors. Microbiota-induced alteration of the production of satiety gastrointestinal peptides, resulting in increased food intake, has been well documented in obesity (review Gomes et al., 2018). The role of the microbiota *per se*, apart from the confounding effect of diet, on satiety (or food intake) is less well documented. In the OP-OR rodent model used in the present study, food/caloric intake has been characterized mostly on males without any association with the composition of gut microbiota. When fed HED, OP rats eat significantly more food than OR rats (Levin, 1999; Ricci and Levin, 2003; Obanda et al., 2018, 2020), but one work showed a higher food intake in OR compared with OP male rats (Levin et al., 1986). In females, only one study showed that before gestation female OP eat and weigh more than OR ones on HED (Frihauf et al., 2016). In the current work, we explored feeding behavior using multiple tests allowing fine-tune analysis of satiety, and, taken together, several items of these tests are moving in the same direction of an altered satiety in F-OP groups.

### What Are the Cues for Crosstalk Between Microbiota and Key Elements of Feeding Behavior?

Our search for associations between bacterial abundances and variation in eating behavior indicators revealed different associations, mainly for abundances quantified at PND21. Although not proving a causal relationship, these associations do support the existence of an impact of certain bacterial populations on eating behavior, which would have occurred mainly in the neonatal period. Such influence could be exerted through different pathways. First, intestinal epithelium development and function could be affected during early stage (Sommer et al., 2015), such as the enteroendocrine cells, which are known to modulate food intake, mainly through gastrointestinal peptide secretion (Martin et al., 2019). Furthermore, early microbiota could influence host metabolism by modulating peripheral organ programming such as the pancreas, liver, adipose tissue, or muscles, also with long-term consequences (Backhed, 2011). In turn, these organs influence food intake *via* endocrine and/or immune and/or vagal routes (McDermott et al., 2006; Abdalla, 2017; Browning et al., 2017). However, in this work, metabolic plasmatic markers (data not shown) were not significantly different between groups, neither at weaning nor at adulthood. The present results tend to rule out the metabolic endocrine route, but a more complete characterization of the energetic regulation is required to conclude on that aspect.

More likely, an early modulation of neurodevelopment which could lead to long-term consequences on brain structure and function may be suspected. Whether early microbiota participates in the development of the central nervous system has been proposed in a series of publications and examined through the observation that key events as microglia proliferation, synaptogenesis, and pruning are impacted in germ-free mice, after antibiotics treatment or after specific fecal transplantation and result in behavioral modifications as anxiety-like symptoms, depression, or autism spectrum disorder (Goyal et al., 2015; Jena et al., 2020; Morais et al., 2020). Food intake regulation is the result of complex interplays between a large number of structures; therefore, to deepen our knowledge on the impact of early microbiota transfer, we need to go further and perform a detailed anatomical, transcriptomic, and functional analysis of the brain structures (VTA, NAc, HT, and NTS) and the different element of the gut–brain communication that can be disturbed according to the existing microbiota.

Since our work is the first to establish the proof of concept that the early intestinal microbiota can influence eating behavior later in life, the mechanistic pathways or mediators relaying the impact of the early microbiota on the central circuitries that regulate eating behavior are obviously unknown. However, they are probably close to those suspected as involved in the interaction between microbiota and neurodevelopment, which rely mainly on the production of specific microbiotal metabolites such as the SCFA or neuromediators and the microbiota-induced mediation of immune signaling (see Warner, 2019 for review).

With this respect, the association we observed in males between the abundance of UBA1819 (a taxon affiliated to the *Faecalibacterium* sp.) which was significantly more abundant in the F-OR group at weaning and the total fat consumption during taste preference tests is of particular interest given the immunomodulatory potential of some species belonging to this genus (Delgado et al., 2020). Similarly, the correlation that we observe for females between the abundance of cluster 10 (affiliated to *Enterobacteriaceae*) at PND21 and *z*-score is intriguing since *E. coli* synthetize dopamine (see Lyte and Lyte, 2019 for review) while this property is present in less than 5% of the bacterial genomes representative of the human intestinal microbiota (Valles-Colomer et al., 2019). Further investigation of these tracks requires more precise bacterial identification, if possible at the strain level, since differences in physiological effects have been observed at this taxonomic scale, particularly in the case of appetite regulation by the intestinal microbiota (Chagwedera et al., 2019). These precisions can be obtained by applying a metagenomic study, which will also make it possible to search for associations with particular functions. Interestingly in this respect, Chagwedera et al. (2019) showed that to be effective, their sole effective strain had to be administered to mice with other microbiotal components but not in pure culture. This feature is most likely related to the fact that a gene coding for a bacteriocin has been identified among the specific genes of this strain. This reminds that correlations do not necessarily reveal direct relations between bacteria and mechanisms supposedly involved in host physiology regulation.

## Conclusion

The current study clearly supports the hypothesis that the transfer of microbiota from mother to pup in early neonatal life can impact eating behavior at adulthood. Our study is the first to go beyond food intake and body weight gain to examine in the same animals and across time the various aspect of eating behavior. Functional metagenomics and offspring metabolomics should help explain how microbiotal activity can alter physiology with regard to eating behavior regulation. Whether mother-to-child microbiotal transfer plays a role in the transmission of metabolic risk clearly deserves further investigation in humans.

## Data Availability Statement

The datasets used and/or analysed during the current study are available from the corresponding author on reasonable request. The 16S rRNA gene sequences analyzed in this study are available through the National Center for Biotechnology Information (NCBI) Sequence Read Archive Dataset under accession number SRP310757/PRJNA714462, accessible with the following link: https://www.ncbi.nlm.nih.gov/sra/PRJNA714462.

## Ethics Statement

The animal study was reviewed and approved by Protocols were approved by the local Committee on the Ethics in Animal Experiments of Pays de la Loire (France) and the French Ministry of Research (APAFIS#6617-2016072916395797-v6). Animal facility is registered by the French Veterinary Department as A44276.

## Author Contributions

PP, CM, GL, CH, and EL designed the research. CM, GL, PP, A-LP, HB, TM, AP, and EL performed the research. PP, CM, GL, and EL supervised the research. CM, GL, PP, A-LP, HB, TM, and EL analyzed the data. PP, CM, GL, and A-LP wrote the manuscript. All authors edited and approved the manuscript.

## Conflict of Interest

The authors declare that the research was conducted in the absence of any commercial or financial relationships that could be construed as a potential conflict of interest.

## Footnotes

1 https://vm-galaxy-prod.toulouse.inra.fr/galaxy/
