# Supplementary material for: Maternal Microbiota Transfer Programs Offspring Eating Behavior
Source: Front Microbiol. 2021 Jun 15;12:672224. doi: 10.3389/fmicb.2021.672224 (PMC8239415; doi:10.3389/fmicb.2021.672224)
Supplement: Supplementary file 1 [file Data_Sheet_1.PDF]

Table S1. Volumes and number of bacteria as determined by qPCR transferred to F344 recipient pups through vaginal, milk-associated and faecal inocula, according to postnatal days (PND). G3: 2-3 days before parturition.

	**F-OP and F-OR**									**F-Sham**
F344 pups Age (PND)	**Vaginal inocula**			**Milk associated inocula**			**Fecal inocula**			**Stock solution**
	Sampled at	µL per pup	Log (Bacteria) per pup	Sampled at	µL per pup	Log (Bacteria) per pup	Sampled at	µL per pup	Log (Bacteria) per pup	µL per pup
PND0	G3	30	5.4±0.3	PND1	10	3.2±0.1	G3	15	7.4±0.4	55
PND1-3				PND1	10	3.2±0.1	PND1	15	7.5±0.3	25
PND4-7				PND5	20	5.4±1.5	PND5	15	7.7±0.3	35
PND8-11				PND10	20	5.5±0.9	PND10	15	7.7±0.2	35
PND12-15				PND15	50	6.6±0.9	PND15	15	8.0±0.5	65

Table S2. Composition of diets.

	Standard Diet (SD)<br>Safe<br>A03	Hyper-energetic diet (HED)<br>TestDiet<br>58V8
**Energy density (kcal/g)**	**3.24**	**4.60**
**Carbohydrate (% energy)**	**61.3**	**35.8**
Carbohydrate (% composition)	59.2	41.2
Carbohydrate composition	*wheat, maize, barley, soya*	*sucrose, maltodextrin, dextrin*
**Protein (% energy)**	**25.2**	**18.1**
Protein (% composition)	24.3	20.8
Protein composition	*Fish, cereals*	*casein*
**Fat (% energy)**	**13.5**	**46.1**
Fat (% composition)	5.8	23.6
Fat composition	*soya*	*lard, soya*
**Fiber (% composition)**	**4.6**	**5.8**
Fiber composition	*bran, soya*	*cellulose powder*

Table S3. Ethogram used to analyze food motivation in the Straight Alley test. *SB*; Starting Box; *GB*: Goal Box

**Behavior code**	**Behavior type**	**Description**	**Excluded behaviors**
Test begin	Point event	Opening of SB	
Leaves	Point event	Rat leaves SB	SB
Arrives	Point event	Rat head is overhanging the food reward at the end of the alley.	
Test end	Point event	Test ends when rat bites the food reward, or after 120 seconds after opening of SB	Still, Moving, SB, Alley, GB
Moving	State event	Rat moves on its four paws (walks, runs, makes an about-turn)	Still
Still	State event	Rat does not move (rearing + stops)	Moving
About-turn	Point event	Rat makes an about-turn	
Rearing	Point event	Rat stops and rears on its back legs to sniff or look at its environment.	
Stop	Point event	Rat stops at least 1 second remaining on its four paws (smell something on the allay floor, freeze, ...)	
SB	State event	Rat is in the SB	Alley, GB
GB	State event	Rat is in the GB	Alley, SB
Alley	State event	Rat is in the alley but not in the SB nor in the GB	SB, GB

Table S4. Behavioural items used to calculate integrated z-score.

Behavioural test	Selected items
Early suckling behavior	Weight gain (g/kg)
Food intake	Daily food intake mean through SD period (kcal/kg/d)<br>Daily food intake mean through first 10 days fed HED (kcal/kg/d)
Liquid taste preference	Total sweet taste intake (g/kg)<br>Total fat taste intake (g/kg)
Food motivation	Ingestion score<br>Inverse of distraction number during the first walk to the Goal Box
Detailed 24 hours meal pattern	Meal number during night phase<br>Mean food intake per meal during night phase (g/kg)
Behavioural Satiety Sequence	Total eat duration (%)<br>Total sleep duration (%)

Table S5. Alpha-diversity indexes calculated for faecal and milk-derived inoculas from OP and OR dams (means ± SD)

	***Faecal inoculas* (all sampling time)**		***Milk-derived inoculas* (all sampling time)**	
	**OP**	**OR**	**OP**	**OR**
**Observed OTUs**	291±46.4	315±32.7	66±16.4	69±13.2
**chao1**	326±35.8	342±29.5	97±30.3	101±25.4
**se.chao1**	14.4±4.9	11.9±3.5	17.6±12.1	17.3±9.3
**ACE**	321±36.4	339±25.5	108.5±39.4	108.4±20.6
**se.ACE**	8.7±0.5	8.9±0.3	5.9±1.8	5.9±0.7
**Simpson**	0.93±0.05	0.95± 0.03	0.62±0.10	0.5±0.14
**Shannon (H’).**	3.67±0.61	3.95± 0.28	1.33±0.27	1.23±0.29
**InvSimpson**	21.2±12.2	23.8±6.7	2.81±0.84	2.39±0.72
**Evenness (H’)**	0.45±0.06	0.48±0.03	0.22±0.04	0.20±0.05
**Evenness (Simpson)**	0.07±0.05	0.05±0.03	0.39±0.10	0.46±0.14

Table S6 : Total bacteria numbers in gut microbiota samples (Log eqbact.g-1of wet weight (medians ± interquartiles)

	F-Sham	F-OP	F-OR	P value Kruskall-Wallis
**PND11**	11.7±0.7	12.0±0.7	12.0±0.5	0.104
**PND21 females**	11.4±0.7	11.8±0.1	11.6±0.5	0.080
**PND21 males**	11.1±0.6	11,6±0.8	11,5±0.5	0.053
**PND60 females**	10.9±0.6	10.9±0.6	10.8±1.1	0.902
**PND60 males**	10.6±0.6	10.7±0.8	10.5±0.5	0.594
**PND130 females**	11.8±1.4	11.5±0.6	11.4±0.9	0.946
**PND130 males**	11.8±0.7	11.7±0.8	11.6±0.6	0.882
**PND200 females**	11.8±0.4	11.9±0.3	11.8±0.4	0.307
**PND200 males**	11.9±0.6	11.9±0.3	11.9±0.4	0.996
