# Supplementary material for: Maternal Microbiota Transfer Programs Offspring Eating Behavior
Source: Front Microbiol. 2021 Jun 15;12:672224. doi: 10.3389/fmicb.2021.672224 (PMC8239415; doi:10.3389/fmicb.2021.672224)
Supplement: Supplementary file 2 [file Data_Sheet_2.PDF]

Supplementary Material and methods

Inocula preparation

Aliquots (400 µL) of vaginal suspensions from OP and OR donors were pooled 2 by 2 for each genotype. Pharmaceutic glycerol (Laboratoire Gilbert, Hérouville-Saint-Clair, France) was added to each pool (10% v/v) and pools were aliquoted and stored in sterile sealed tube at -80°C until transfer to pups. The residual volumes (approx. 130 µL) of each pool (3 from OP and 3 from OR) were centrifuged (15 min, 5400g, 4°C) then pellets were stored at -20°C in sterile sealed tube for further extraction of bacterial DNA.

At each stage of lactation (Figure 1A), fresh raw milk was diluted twice with sterile citrate buffer 2% (w/v) in order to emulsify lipids. An hundred microliters of these suspensions were plated of Wilkins-Chalgren agar and incubated at 37°C for 48h in anaerobic conditions (GENbox Jar 7.0L Biomérieux, Marcy l'Etoile, France and BD GasPack EZ Anaerobe, Becton Dickinson & Company, Sparks, Maryland, USA). Colonies from the first dilution were suspended twice in 2x1mL of sterile NaCl 0.9% with a sterile spreader (Biologix, Chicago, Illinois, USA) and the suspensions were harvested, pooled and centrifuged (15min, 5400g, 4°C). Pellets were suspended in 1mL of sterile NaCl 0.9% then pooled according to the same couples of dams as for the vaginal inocula. An aliquot (300µL) of each of these pooled suspensions was centrifuged (15min, 5400g, 4°C) and pellet was stored at -20°C for bacterial DNA extraction. The remaining pooled suspensions were 1000-fold diluted in sterile stock solution (NaCl 0.9% added with glycerol 10% (v/v)), aliquoted and stored in sterile sealed tube at -80°C until transfer to pupsAt each stage of lactation (Figure 1A), fresh raw milk was diluted twice with sterile citrate buffer 2% (w/v) in order to emulsify lipids. Aliquots (100µL) of these suspensions were serially diluted four times in sterile NaCl 0.9% and plates of Wilkins-Chalgren agar were then inoculated for each dilution

and incubated at 37°C for 48h in anaerobic conditions (GENbox Jar 7.0L Biomérieux, Marcy l'Etoile, France and BD GasPack EZ Anaerobe, Becton, Dickinson & Company, Sparks, Maryland, USA). Colonies from the first dilution were suspended twice in 2x1mL of sterile NaCl 0.9% with a sterile spreader (Biologix, Chicago, Illinois, USA) and the suspensions were harvested, pooled and centrifuged (15min, 5400g, 4°C). Pellets were suspended in 1mL of sterile NaCl 0.9% then pooled according to the same couples of dams as for the vaginal inocula. An aliquot (300µL) of each of these pooled suspensions was centrifuged (15min, 5400g, 4°C) and pellet was stored at -20°C for bacterial DNA extraction. The remaining pooled suspensions were 1000-fold diluted in sterile stock solution (NaCl 0.9% added with glycerol 10% (v/v)), aliquoted and stored in sterile sealed tube at -80°C until transfer to pups.

For each harvesting time, faeces were pooled according to the same couples of dams as for other inocula. Pooled fecal samples (2x100mg) were homogenized at 5000rpm (2x15s intervals with 10s break) in 3mL sterile stock solution plus L-Cystein 1% (Sigma-Aldrich, France) using a Precellys® "evolution" bead-beater (Bertin, Montigny-le-Bretonneux, France). Faecal pools suspensions were then filtered using sterile gauze (Laboratoire Urgo, Chenôve, France) and aliquoted before storage at -80°C. An aliquot (0.3 mL) of the remaining volume was centrifuged (15min, 5400g, 4°C) and pellets were stored at -20°C for extraction of bacterial DNA.

DNA extraction

DNA extraction from vaginal inocula

DNA was extracted from vaginal inocula using a protocol adapted from (Neuendorf, Gajer et al. 2015) using the QIAmp Fast DNA Mini kit (Qiagen, Hilden, Germany). Pellets were suspended in 0.5 mL NaCl 0.9% and incubated 30 min at 37°C with a mix of lysozyme (50µg/mL) and mutanolysin (178 U/mL) (all from Sigma-Aldrich, Saint-Quentin Fallavier,

France) in 0.5 mL PBS. Then a mix of proteinase K (0.2 mg/mL) from QIAmp Fast DNA kit, SDS 0.5%, DNAse- and protease-free RNAse A (0.02mg/mL) (Fisher Scientific, Illkirch, France) was added (45 min, 55°C). Mechanical lysis was performed using a Precellys® "Evolution" bead-beater during 40s at 7400rpm in 100mg Zirconium powder beads (0.1mm) (BioSpec products, VWR International, Fontenay-sous-Bois, France). The lysates were processed according to the QIAmp Fast DNA Mini kit protocol (Qiagen, Hilden, Germany). DNA was eluted with 50µL of AE buffer after 5min incubation at room temperature and quantified with NanoVue (GE Healthcare, Illinois, USA) (between 0.6 and 1.5ng/µL) before storage at -20°C.

DNA extraction from milk inocula

DNA was extracted from milk inocula pellets using QIAmp Fast DNA Mini kit (Qiagen, Hilden, Germany) with additional lysis steps adapted to the protocol from (Neuendorf, Gajer et al. 2015). Pellets were suspended in 300µL-lysis buffer (1h, 37°C) containing lysozyme (20mg/mL final), Tritonx100 (1.2% final), Tris-HCl (20mM,), EDTA (2mM), lysostaphin (46U/mL) and mutanolysin (577 U/mL). Mechanical disruption was performed using 5 Zirconium sterile beads (2mm) (Fischer Scientific, Illkrich, France) and a Precellys® "Evolution" bead-beater (3x20s, 5600g), which was followed by thermal lysis at 95°C for 5min DNA- and protease-free RNAse A (50µg, Fisher Scientific, Illkrich, France) was then added at RT (15min) followed by incubation (30 min, 56°C) with proteinase K (25µL) in 300 µL AL buffer (both from QIAmp Kit). After centrifugation (2min at 13000g), supernatants were collected in new sterile tubes containing 600µL of absolute ethanol and loaded on kit columns. DNA was eluted with 100µL of QIAmp kit AE buffer.

DNA extraction from faecal & intestinal samples

DNA was extracted from fecal or caeco(colonic) pellets using QIAmp Fast DNA Stool Mini kit (Qiagen, Hilden, Germany) after enzymatic and mechanical disruptions as described previously (Le Drean, Pocheron et al. 2019) except that RNA digestion using DNase- and protease-free RNase A (Fisher Scientific, Illkrich, France) was carried out before the proteinase K digestion step d*escribed in the manufacturer's instructions.*

16SrDNA sequences processing

Reads processing was performed with the FROGS v3 pipeline (Escudie, Auer et al. 2018) in Galaxy environment (https://vmgalaxy-prod.toulouse.inra.fr/galaxy/). Reads were merged using VSearch (Rognes, Flouri et al. 2016) with 10% mismatches authorized in overlapped region of 50pb minimum (amplicon size of 100pb to 450pb). Primers sequences were removed using (Martin 2011) with 10% mismatches authorized. Merged reads were clustered using Swarm (Mahe, Rognes et al. 2014) according to (Escudie, Auer et al. 2018) recommendations, with a first step with an aggregation parameter equal to 1, followed by a second step on previous clusters seeds with an aggregation parameter equal to 3. Chimera detection and removal was performed using VSearch. Clusters were then filtered on abundance with a minimum 0.005% of all sequences and affiliated using blastn+ (Camacho, Coulouris et al. 2009) against 16S Silva v.132 database (Quast, Pruesse et al. 2013) with a pintail of 80. Final raw abundances ranged from 16,567 to 86,405 sequences with the minimal value observed for a faecal sample collected at PND60 and the maximal value results from a caecocolonic content harvested at PND21. Using FROGS rarefaction tool, counts were then rarefied to 16,567 reads per sample, a threshold which matched satisfactorily with the levelings of the rarefaction curves (data not shown).

Final raw abundances ranged from 16,567 to 95,556 sequences. Counts were then rarefied to 16,567 reads per sample using FROGS rarefaction tool. Alpha- and beta-diversity

indexes were calculated on raw or rarefied (respectively) abundances using Vegan *(v*2.5-5) and Phyloseq packages (v1.28.0, (McMurdie and Holmes 2013) and R (*v.3.6.1*).

Camacho, C., G. Coulouris, V. Avagyan, N. Ma, J. Papadopoulos, K. Bealer and T. L. Madden (2009). "BLAST+: architecture and applications." BMC Bioinformatics **10**: 421.

Escudie, F., L. Auer, M. Bernard, M. Mariadassou, L. Cauquil, K. Vidal, S. Maman, G. Hernandez-Raquet, S. Combes and G. Pascal (2018). "FROGS: Find, Rapidly, OTUs with Galaxy Solution." Bioinformatics **34**(8): 1287-1294.

Le Drean, G., A. L. Pocheron, H. Billard, I. Grit, A. Pagniez, P. Parnet, E. Chappuis, M. Rolli-Derkinderen and C. Michel (2019). "Neonatal Consumption of Oligosaccharides Greatly Increases L-Cell Density without Significant Consequence for Adult Eating Behavior." Nutrients **11**(9).

Mahe, F., T. Rognes, C. Quince, C. de Vargas and M. Dunthorn (2014). "Swarm: robust and fast clustering method for amplicon-based studies." PeerJ **2**: e593.

Martin, M. (2011). "Cutadapt removes adapter sequences from high-throughput sequencing reads. ." EMBnet. journal **17**(1): 10-12.

McMurdie, P. J. and S. Holmes (2013). "phyloseq: an R package for reproducible interactive analysis and graphics of microbiome census data." PLoS One **8**(4): e61217.

Neuendorf, E., P. Gajer, A. K. Bowlin, P. X. Marques, B. Ma, H. Yang, L. Fu, M. S. Humphrys, L. J. Forney, G. S. Myers, P. M. Bavoil, R. G. Rank and J. Ravel (2015). "Chlamydia caviae infection alters abundance but not composition of the guinea pig vaginal microbiota." Pathog Dis **73**(4).

Quast, C., E. Pruesse, P. Yilmaz, J. Gerken, T. Schweer, P. Yarza, J. Peplies and F. O. Glockner (2013). "The SILVA ribosomal RNA gene database project: improved data processing and web-based tools." Nucleic Acids Res **41**(Database issue): D590-596.

Rognes, T., T. Flouri, B. Nichols, C. Quince and F. Mahe (2016). "VSEARCH: a versatile open source tool for metagenomics." PeerJ **4**: e2584.
